# Molecular magnetic resonance imaging of myeloperoxidase activity identifies culprit lesions and predicts future atherothrombosis

**DOI:** 10.1093/ehjimp/qyae004

**Published:** 2024-01-24

**Authors:** James Nadel, Xiaoying Wang, Prakash Saha, André Bongers, Sergey Tumanov, Nicola Giannotti, Weiyu Chen, Niv Vigder, Mohammed M Chowdhury, Gastao Lima da Cruz, Carlos Velasco, Claudia Prieto, Andrew Jabbour, René M Botnar, Roland Stocker, Alkystis Phinikaridou

**Affiliations:** Heart Research Institute, Arterial Inflammation and Redox Biology Group, 7 Eliza St, Newtown, Sydney, NSW 2042, Australia; Department of Cardiology, St Vincent’s Hospital, Sydney, NSW, Australia; Department of Medicine and Health, University of New South Wales, Sydney, NSW, Australia; School of Biomedical Engineering & Imaging Sciences, King’s College London, London, UK; Academic Department of Surgery, Cardiovascular Division, King’s College London, London, UK; Biological Resources Imaging Laboratory, University of New South Wales, Sydney, NSW, Australia; Heart Research Institute, Arterial Inflammation and Redox Biology Group, 7 Eliza St, Newtown, Sydney, NSW 2042, Australia; Faculty of Medicine and Health, The University of Sydney, Sydney, NSW, Australia; Medical Imaging Science, Faculty of Medicine and Health, The University of Sydney, Sydney, NSW, Australia; Heart Research Institute, Arterial Inflammation and Redox Biology Group, 7 Eliza St, Newtown, Sydney, NSW 2042, Australia; Heart Research Institute, Arterial Inflammation and Redox Biology Group, 7 Eliza St, Newtown, Sydney, NSW 2042, Australia; Department of Vascular Surgery, University of Cambridge, Cambridge, UK; Department of Radiology, University of Michigan, Ann Arbor, USA; School of Biomedical Engineering & Imaging Sciences, King’s College London, London, UK; School of Biomedical Engineering & Imaging Sciences, King’s College London, London, UK; Pontificia Universidad Católica de Chile, Institute for Biological and Medical Engineering, Santiago, Chile; Department of Cardiology, St Vincent’s Hospital, Sydney, NSW, Australia; Department of Medicine and Health, University of New South Wales, Sydney, NSW, Australia; School of Biomedical Engineering & Imaging Sciences, King’s College London, London, UK; Pontificia Universidad Católica de Chile, Institute for Biological and Medical Engineering, Santiago, Chile; King’s BHF Centre of Research Excellence, London, UK; Heart Research Institute, Arterial Inflammation and Redox Biology Group, 7 Eliza St, Newtown, Sydney, NSW 2042, Australia; School of Biomedical Engineering & Imaging Sciences, King’s College London, London, UK; King’s BHF Centre of Research Excellence, London, UK

**Keywords:** atherosclerosis, atherothrombosis, carotid endarterectomy, molecular magnetic resonance imaging, myeloperoxidase, plaque disruption

## Abstract

**Aims:**

Unstable atherosclerotic plaques have increased activity of myeloperoxidase (MPO). We examined whether molecular magnetic resonance imaging (MRI) of intraplaque MPO activity predicts future atherothrombosis in rabbits and correlates with ruptured human atheroma.

**Methods and results:**

Plaque MPO activity was assessed *in vivo* in rabbits (*n* = 12) using the MPO-gadolinium (Gd) probe at 8 and 12 weeks after induction of atherosclerosis and before pharmacological triggering of atherothrombosis. Excised plaques were used to confirm MPO activity by liquid chromatography–tandem mass spectrometry (LC–MSMS) and to determine MPO distribution by histology. MPO activity was higher in plaques that caused post-trigger atherothrombosis than plaques that did not. Among the *in vivo* MRI metrics, the plaques’ R1 relaxation rate after administration of MPO-Gd was the best predictor of atherothrombosis. MPO activity measured in human carotid endarterectomy specimens (*n* = 30) by MPO-Gd–enhanced MRI was correlated with *in vivo* patient MRI and histological plaque phenotyping, as well as LC–MSMS. MPO-Gd retention measured as the change in R1 relaxation from baseline was significantly greater in histologic and MRI-graded American Heart Association (AHA) type VI than type III–V plaques. This association was confirmed by comparing AHA grade to MPO activity determined by LC–MSMS.

**Conclusion:**

We show that elevated intraplaque MPO activity detected by molecular MRI employing MPO-Gd predicts future atherothrombosis in a rabbit model and detects ruptured human atheroma, strengthening the translational potential of this approach to prospectively detect high-risk atherosclerosis.

## Graphical Abstract

**Graphical Abstract:**
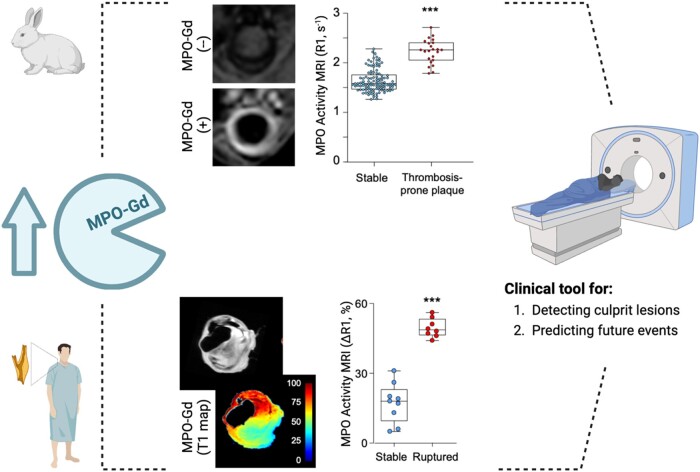
Molecular magnetic resonance imaging of myeloperoxidase activity selectively predicts future atherothrombosis in a preclinical model and detects ruptured human atheroma.

## Introduction

Atherosclerosis remains the main cause of morbidity and mortality in the western world.^1^ Current non-invasive clinical imaging techniques have improved the management and treatment of atherosclerosis by identifying total plaque burden and severity of luminal stenosis.^2^ Nevertheless, stenosis alone is a poor predictor of future cardiovascular events, and the benefit of intervention based on luminal narrowing alone is limited.^3–5^ Rather, overwhelming evidence shows that plaque composition and biological activity are superior determinants of acute events.^6,7^ Unfortunately, current imaging strategies provide only surrogate markers of plaque instability or disease activity based on structural determinants^8,9^ of plaque composition or non-specific imaging probes.^10^

Vascular inflammation is a key driver for destabilization and rupture/erosion of atherosclerotic plaques.^11,12^ Although imaging of vascular inflammation and tracking the anti-inflammatory effects of therapeutics have been achieved using magnetic resonance imaging (MRI),^13–15^ positron emission tomography (PET),^16,17^ and computed tomography (CT),^18^ there are currently no clinically available imaging agents that can specifically differentiate between disease-promoting and disease-reparative inflammation.

The inflammatory enzyme myeloperoxidase (MPO) generates highly reactive hypochlorite within the phagolysosome as part of the innate immune response.^19^ However, up to 30% of neutrophil MPO can be released into the extracellular space, where it causes tissue injury, including atherosclerotic plaque destabilization.^20–23^ Molecular MRI of MPO activity has been achieved using the MPO-selective probe [gadolinium-*bis*-5-hydroxytryptamide diethylenetriaminepentaacetic acid (MPO-Gd)].^24^ MPO-mediated oxidation of the phenol group of the 5-hydroxytryptamide moiety forms MPO-Gd radicals that give rise to probe oligomerization and probe–protein adduct formation. This increases the probe’s relaxivity and retention in tissues with elevated MPO activity giving rise to enhanced MRI signal. MPO-Gd MRI was successfully used to image inflammation in rabbits with atherosclerosis.^25^ Moreover, in an animal model of plaque instability, MPO activity was higher in histologically unstable than stable plaque; MPO-Gd retention determined by molecular MRI correlated with plaque destabilization; and genetic or pharmacological blockade of MPO activity increased plaque stability,^26,27^ thus establishing MPO as causal to plaque destabilization.

Despite these promising preclinical results, the value of quantitative/direct measure of plaque MPO activity to non-invasively predict clinically more relevant plaque disruption and thrombosis remains unknown. We hypothesized that increased plaque MPO activity predicts plaque destabilization and ensuing thrombosis, and that MPO-Gd enables non-invasive detection of ruptured atherosclerotic lesions. We tested this hypothesis by assessing MPO activity using MPO-Gd-enhanced MRI and a validated method based on liquid chromatography–tandem mass spectrometry (LC–MSMS)^28,29^ in a rabbit model of controlled atherothrombosis and in human plaques.

## Methods

An established rabbit model of atherothrombosis was used for *in vivo* MRI with MPO-Gd.^30,31^ Lesion-free arteries as well as stable and thrombosis-prone plaques were excised and examined for MPO activity by LC–MSMS and MPO protein by immunohistochemistry, and disease outcome related to MPO activity. MPO activity in human carotid endarterectomy (CEA) specimens was evaluated by *ex vivo* MPO-Gd-enhanced MRI, as well as LC–MSMS. MPO activity was associated with plaque phenotypes determined by both *in vivo* pre-surgical MRI and histology. Detailed materials and methods of this study are available in the Supplementary material.

## Results

### Molecular imaging of arterial MPO activity predicts trigger-induced atherothrombosis

All 12 rabbits developed atherosclerosis, and 5 advanced to trigger-induced atherothrombosis (see Supplementary data online, *Figure S1A*). A total of 236 aortic segments were analysed pre-trigger using 2D T_1_-weighted black blood (T1BB), 3D inversion recovery T1w (T1w-IR), and 3D T_1_ mapping images. These segments were characterized as lesion-free artery (*n* = 87) or containing plaques (*n* = 149) of various disease stages and trigger-induced outcomes (see Supplementary data online, *Figures S1B* and *C* and *S2*). Of the 149 plaque-containing segments, 24 (16%) developed trigger-induced thrombosis with the remaining 125 (84%) categorized as thrombosis-resistant, stable plaques.

**Figure 1:**
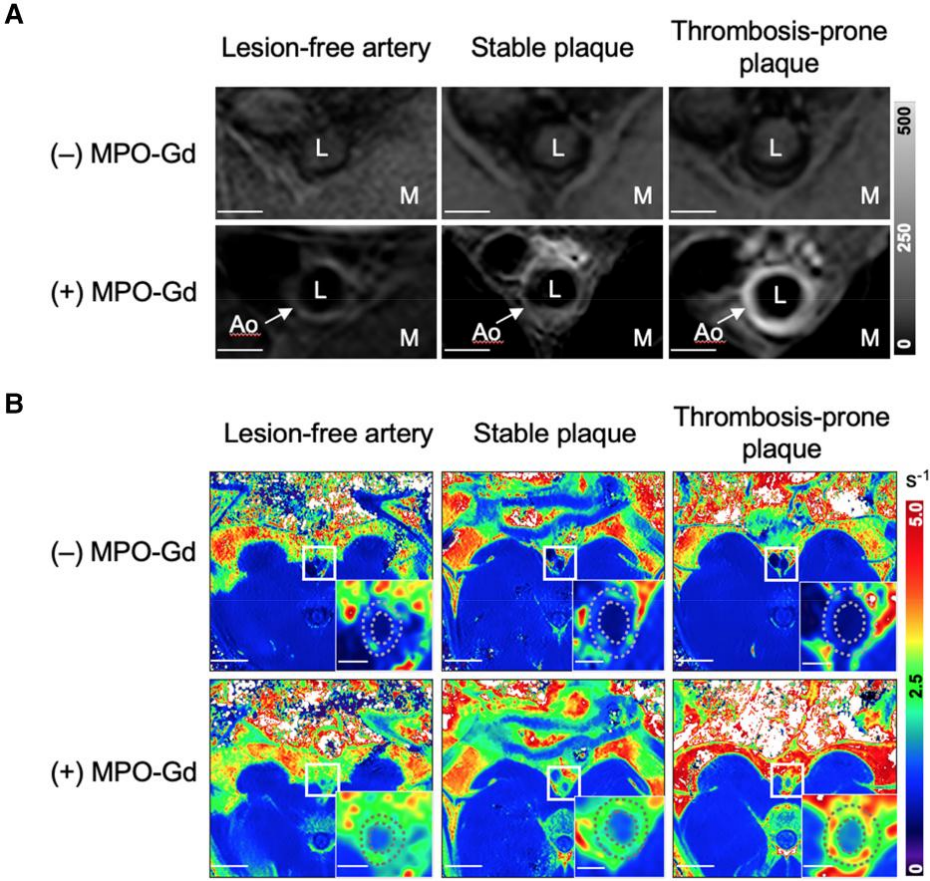
*In vivo* pre-trigger MR images of rabbit aortic segments 12 weeks after induction of atherosclerosis. (*A*) T1w inversion recovery images before and 1 h after injection of MPO-Gd. Scale bar 2 mm. (*B*) R1 relaxation maps before and 1.5 h after injection of MPO-Gd. Scale bars are 20 and 2 mm for main panels and insets, respectively. Ao, aortic wall; L, aortic lumen; M, paraspinal muscle.

Representative *in vivo* rabbit MR images from the three groups of aortic segments acquired at Week 12 are shown in *Figure 1*, with the corresponding Week 8 images shown in Supplementary data online, *Figure S3*. Prior to MPO-Gd administration, T1w-IR images showed very low aortic signal intensity (*Figure 1A*), with comparable R1 values for the aortic wall and paraspinal muscle (*Figure 1B*). Late MPO-Gd enhancement of patchy appearance was observed in all three groups, although plaque-containing segments had higher signal intensity and R1 values compared with lesion-free segments.

**Figure 2:**
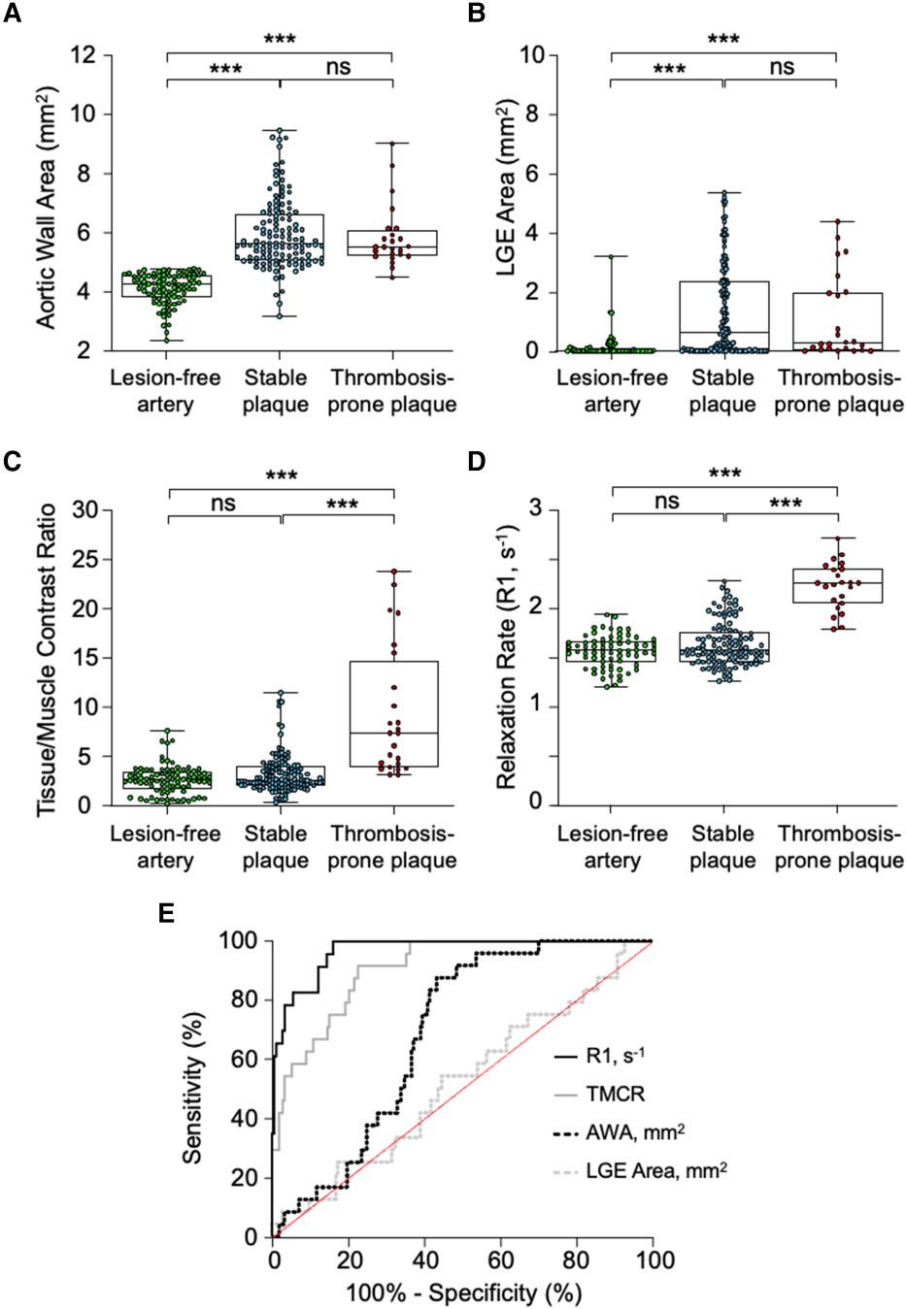
Quantitative analysis of MPO-Gd-enhanced *in vivo* pre-trigger MR images in rabbits 12 weeks after induction of atherosclerosis. (*A*) Aortic wall area (AWA) segmented from T1BB images. (*B*, *C*) Late MPO-Gd-enhanced (LGE) area and aortic tissue-to-muscle contrast ratio (TMCR) 1 h after MPO-Gd administration using T1w-IR images. (*D*) R1 relaxation rate maps 1.5 h after MPO-Gd administration. (*E*) Receiver operating characteristic (ROC) curves of the MRI metrics in predicting trigger-induced thrombosis. ***P < 0.001 and ns > 0.05.

Quantitative MRI analysis of the Week 12 pre-trigger images showed similar aortic wall areas (*P* > 0.9) and late MPO-Gd-enhanced area (*P* = 0.2) in stable and thrombosis-prone plaques (*Figure 2A* and *B*). However, thrombosis-prone plaques had significantly higher R1 values than stable plaques (2.2 ± 0.2 vs. 1.6 ± 0.2 s^−1^, *P* < 0.0001; *Figure 2C* and *D*), while stable plaques and lesion-free aortic segments had comparable R1 values (*P* = 0.5). A similar pattern was observed at 8 weeks (see Supplementary data online, *Table S1*, *Figure S3*).

Receiver operating characteristic (ROC) curves demonstrated that following the administration of MPO-Gd, the pre-trigger R1 value was the best predictor for trigger-induced atherothrombosis with high sensitivity (100%) and specificity (86%), and by comparison, aortic wall area and late MPO-Gd-enhanced area were poor indicators for trigger-induced atherothrombosis (*Figure 2E*; Supplementary data online, *Table S2, Figure S4*).

### Increased MPO activity in thrombosis-prone rabbit plaques

**Figure 3:**
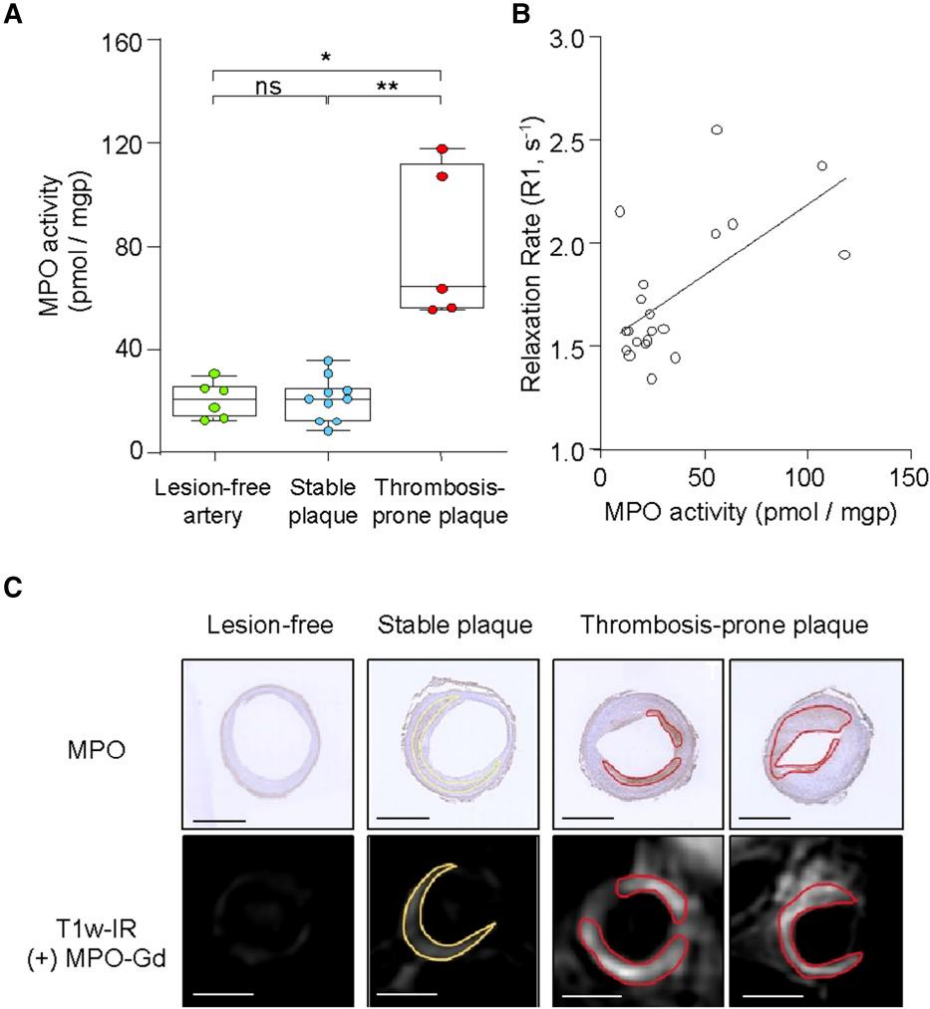
*Ex vivo* plaque MPO activity and correlation with *in vivo* MRI in rabbits. (*A*) Aortic segments containing thrombosis-prone plaques have increased MPO activity. (*B*) Arterial MPO activity measured *ex vivo* correlates with that measured by MRI. (*C*) MPO protein assessed by immunohistochemistry co-localizes with late MPO-Gd enhancement. Scale bars 2 mm. *P < 0.05, **P < 0.01 and ns > 0.05.

We next validated the results obtained with *in vivo* molecular MRI by quantifying the MPO-specific product 2-chloroethidium (2-Cl-E^+^) from added hydroethidine by LC–MSMS *ex vivo*. MPO activity was significantly higher in thrombosis-prone than stable plaques (80.4 ± 30.0 vs. 20.9 ± 8.4 pmol/mgp, *P* = 0.004) and lesion-free aortic segments (20.7 ± 6.9 pmol/mgp, *P* = 0.02; *Figure 3A*). Moreover, MPO activity determined by LC–MSMS showed a positive correlation with R1 values determined from the *in vivo* T_1_ maps (*r* = 0.62; *Figure 3B*). Finally, immunohistochemistry (*Figure 3C*) showed that MPO protein was more abundant in thrombosis-prone than stable plaques, with some/low expression in the media of lesion-free aortic sections. Areas of high MPO expression co-localized with the spatial distribution of MPO-Gd enhancement seen on pre-trigger T1w-IR images.

### MPO-Gd activity as a marker of ruptured plaques in humans

Demographics and characteristics of the clinical study population are presented in Supplementary data online, *Table S3*. Patients were predominantly older Caucasian males on optimal medical therapy, with lipid profiles within guideline-directed ranges. Forty per cent of the cohort had symptomatic carotid disease with 30% having a confirmed ipsilateral stroke on pre-surgical neuroimaging. *In vivo* carotid MRI data are presented in Supplementary data online, *Table S4*. Twelve out of 30 patients underwent dedicated carotid MRI for plaque characterization prior to CEA (see Supplementary data online, *Figure S5*), with most having severe carotid stenoses by NASCET criteria. Most slices analysed by MRI were mature American Heart Association (AHA) type V lesions, and 17% had features of AHA type VI ruptured and destabilized plaques. Interobserver discordance occurred with 24 out of 101 slices assigned an MRI-based AHA grade.

**Figure 4:**
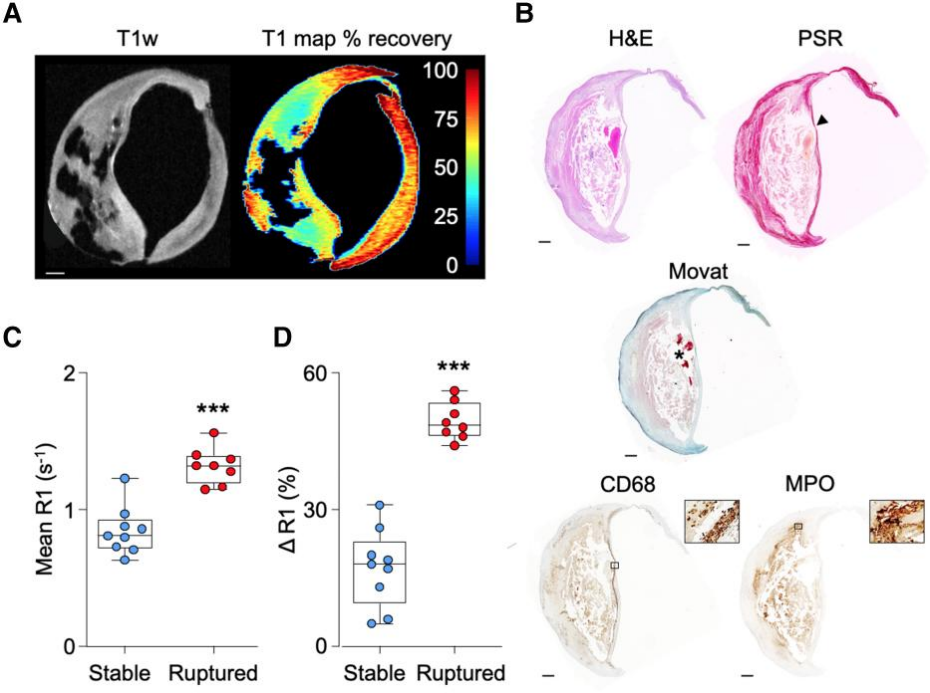
MPO activity assessed by *ex vivo* MPO-Gd retention and AHA grading determined by histology. (*A*) Baseline T1w and T_1_ map of percentage recovery from baseline following MPO-Gd probe activation. Scale bar 1 mm. (*B*) Corresponding histology showing a thin-capped (arrowhead) fibroatheroma with underlying IPH (asterisk), MPO, and CD68-positive staining (magnified boxes ×100). Scale bar 500 μm. Areas of MPO-Gd probe retention correspond to MPO protein determined by immunohistochemistry particularly in the shoulder regions of the plaque. (*C*, *D*) Plots showing higher R1 relaxation rate in ruptured than stable plaques following MPO-Gd activation. ***P < 0.001.

**Figure 5:**
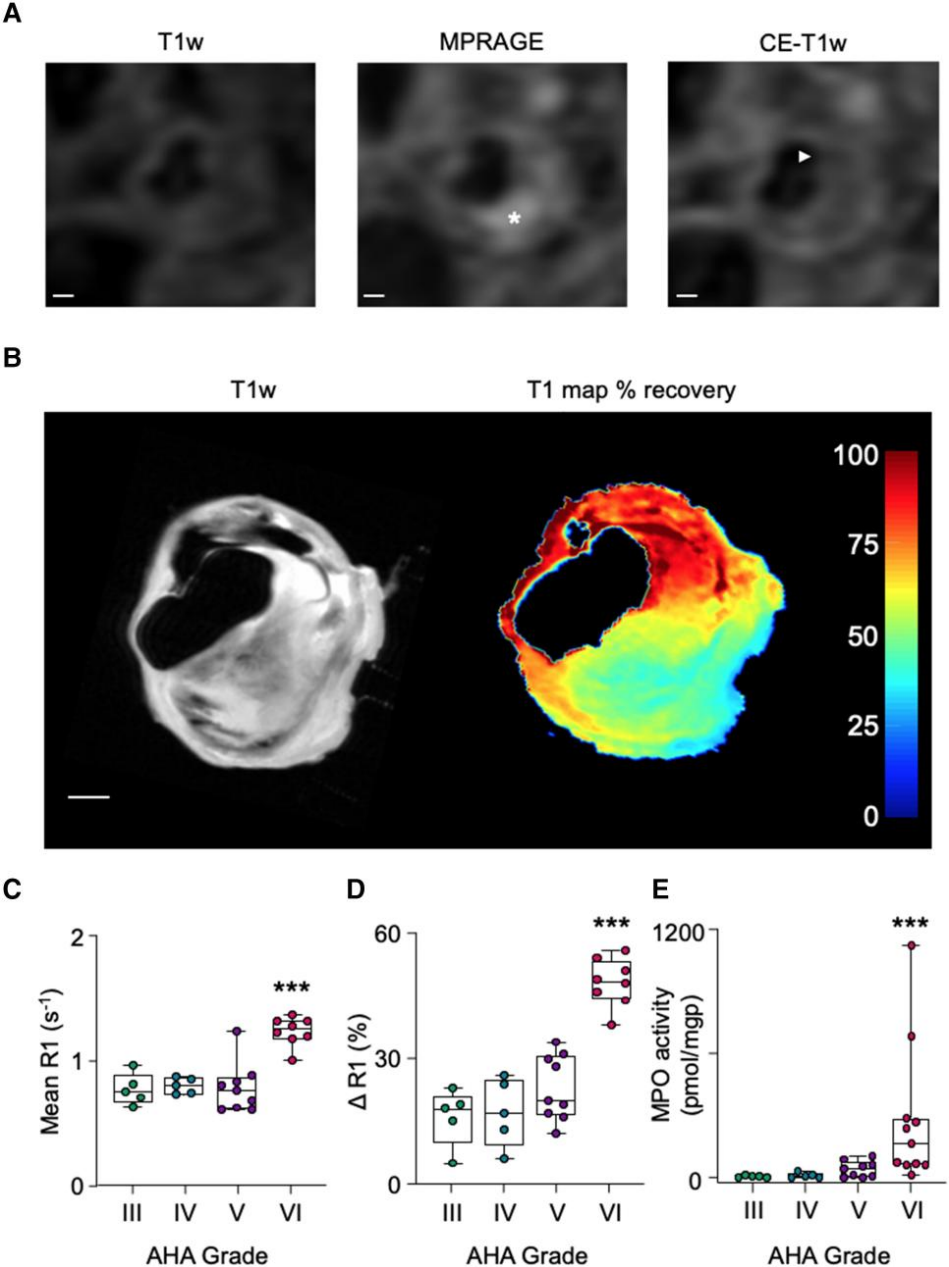
Comparison between AHA grading determined by *in vivo* MRI and MPO activity assessed by *ex vivo* MPO-Gd retention and by LC–MSMS. (*A*) *In vivo* carotid MRI shows a lipid-rich plaque with hyperintense IPH (asterisk) and fibrous cap thinning (arrowhead). (*B*) *Ex vivo* T1w and T_1_ map of percentage recovery from baseline following MPO-Gd probe activation. Probe retention corresponds with the region of hyperintensity seen *in vivo*. Scale bars 1 mm. (*C*, *D*) Following MPO-Gd activation, ruptured/destabilized type VI plaques have higher mean R1 and higher ΔR1 than type III–V plaques. (*E*) Type VI plaques determined histologically using AHA criteria have higher MPO activity than type III–V plaques. ***P < 0.001.

Following optimization of the *ex vivo* molecular MRI method using MPO-Gd (see Supplementary material and Supplementary data online, *Figure S6*), R1 relaxation rates relative to pre-contrast values were deemed to be a reliable surrogate of specific MPO-Gd retention in CEA sections 4 h after probe activation (see Supplementary data online, *Figure S7*). First, T_1_ maps to quantify MPO-Gd retention/MPO activity were compared with histological features (*Figure 4A* and *B*). Areas of MPO-Gd retention co-localized to immunohistochemical MPO staining, particularly in the shoulder regions of plaques. MPO activity was higher in CEA specimens that contained ruptured than stable plaques, as determined by the R1 relaxation rate (1.3 ± 0.1 vs. 0.8 ± 0.2 s^−1^, *P* < 0.0001) and ΔR1 values from baseline (49 ± 4 vs. 17 ± 9%, *P* < 0.0001; *Figure 4C* and *D*). Similarly, when comparing *ex vivo* MPO-Gd retention in whole CEA samples to *in vivo* MRI-determined AHA grade, probe retention corresponded to regions of MPRAGE hyperintensity and cap disruption in slices containing destabilized plaques (*Figure 5A* and *B*). Following MPO-Gd activation, ruptured/destabilized type VI plaques had significantly higher R1 values (1.2 ± 0.1 s^−1^) than type III, IV, and V plaques (0.8 ± 0.1, 0.8 ± 0.1, and 0.8 ± 0.2 s^−1^, respectively; *P* < 0.0001; *Figure 5C*). Similarly, type VI plaques had higher ΔR1 (48 ± 6%) than type III–V plaques (16 ± 7, 17 ± 8, and 2 3 ± 8%, respectively; *P* < 0.0001; *Figure 5D*), indicative of increased MPO activity in ruptured/destabilized plaques. MPO-Gd retention was similar for histologically stable (ΔR1 = 17 ± 9%, *n* = 9) and MRI-graded type III–V (ΔR1 = 20 ± 8%, *n* = 19) plaques (*P* = 0.8) and for histologically ruptured (49 ± 4%, *n* = 8) and MRI-graded type VI (48 ± 6%, *n* = 8) plaques (*P* = 0.6).

The validity of MPO-Gd to detect MPO activity in CEA sections was confirmed by *ex vivo* LC–MSMS quantification of 2-Cl-E^+^. MPO activity was significantly higher in type VI plaques compared with histologically defined type III–V plaques (277 ± 338 vs. 7 ± 6, 11 ± 12, and 42 ± 39 pmol/mgp, respectively; *P* = 0.0008; *Figure 5E*).

## Discussion

We sought to determine the utility of MPO activity quantified by *in vivo* molecular MRI in predicting atherothrombosis and detecting plaque rupture. The results establish for the first time that elevated intraplaque MPO activity detected by MPO-Gd discriminates between thrombosis-prone and stable plaques and predicts future plaque disruption and atherothrombosis in a rabbit model. The data also demonstrate increased MPO activity in ruptured compared with stable human atherosclerotic plaques and that this can be detected by molecular MRI. Our results are the first to demonstrate that molecular MRI of intraplaque MPO activity detects culprit lesions and can predict future susceptibility to atherothrombosis and identify MPO activity as a promising non-invasive strategy for the detection of high-risk plaque.

Our results show that molecular MRI with MPO-Gd has a high diagnostic value in selectively and prospectively detecting plaques that cause thrombosis. This suggests that non-invasive imaging of plaque MPO activity is a promising candidate for clinical translation to predict adverse prognosis and guide treatment. This is supported by several lines of evidence. Arterial MPO activity quantified *in vivo* by R1 relaxation rates reliably predicted trigger-induced plaque disruption with high sensitivity and specificity, and it positively correlated with MPO activity measured by LC–MSMS. Immunohistochemistry showed that MPO protein co-localized with sites of elevated MPO activity in thrombosis-prone plaque. Strikingly, MPO activity was significantly elevated in thrombosis-prone plaques as early as 8 weeks and remained high at 12 weeks. This suggests that plaque MPO activity may be an early diagnostic marker of plaque instability. The results in the rabbit model agree with and extend previous findings in animal models showing that MPO-Gd MRI can detect inflamed atherosclerotic plaques in rabbits^27^ and selectively enhance histologically unstable plaque in mice.^28,29^ Importantly, however, these previous studies employed models in which plaques do not disrupt to cause thrombosis, unlike the situation in the present study and as is seen in most human cases resulting in cardiac mortality.

Animal models that spontaneously develop plaque disruption leading to thrombosis are lacking. In the rabbit model used in the present study, plaque disruption is pharmacologically ‘triggered’ depending on an unstable plaque phenotype and is caused by both plaque rupture and erosion,^30,31^ thereby resembling atherothrombosis in humans.^6,7^ As such, the rabbit model offers the unique opportunity to image plaque activity at precise time points prior to ‘triggering’ atherothrombosis, making it clinically relevant and suitable to develop and test imaging strategies for the detection of unstable plaque prior to cardiovascular events.

Three separate lines of evidence support our notion of increased MPO activity in human ruptured plaque. First, using a highly specific LC–MSMS method to directly determine the chlorinating activity that is specific for MPO,^28^ we show that MPO activity is increased selectively in lesions with fissure, intraplaque haemorrhage (IPH), and thrombus. Second, MPO activity determined by *ex vivo* MPO-Gd retention resulted in R1 relaxation values significantly higher in ruptured compared with stable plaques, the phenotype of which was confirmed by histology. Third, *ex vivo* MPO-Gd retention correlated with AHA plaque classification on *in vivo* carotid MRI, MPO localization, and histologically determined vulnerable plaque features such as IPH and cap disruption/thinning. These observations extend the recent report of increased MPO activity in human plaques at risk of rupture^23^ to culprit lesions and demonstrate the potential clinical application of MPO activity by molecular imaging.

There has been continued effort to establish non-invasive imaging strategies to image inflammatory plaque activity that would enable reliable detection of high-risk plaque.^32–35^ Plaque enhancement with non-targeted MRI contrast agents associates with acute cardiovascular events, and the dynamics of the contrast agent uptake correlates with plaque inflammation.^36^ Nevertheless, enhancement can be influenced by other factors that drive uptake of non-targeted contrast, such as plaque vascularity, and it is therefore not specific to inflammation. Alternatively, ultra-small superparamagnetic iron oxide (USPIO), a clinically approved MRI contrast agent, can track and characterize macrophage infiltration.^14,37^ However, USPIO tends to accumulate in anti-inflammatory macrophages, which compromises its ability to detect detrimental inflammation.^38^ Moreover, unlike Gd-based MRI agents that can be imaged within 1 h of administration, the typical 12–24-h time gap between pre- and post-contrast injection MRI required for USPIO is impractical clinically and may preclude the possibility of short-term follow-up with other diagnostic MRI sequences.^39^

PET tracers including ^18^F-fluorodeoxyglucose (^18^F-FDG) and ^68^Ga-DOTATATE have also been used to image inflammation. ^18^F-FDG-PET targets enhanced glucose uptake and can detect plaque inflammation driven by the accumulation of macrophages. However, it is non-specific,^16^ susceptible to changes in blood glucose concentrations,^40^ generates signals with high heterogeneity,^41^ and cannot differentiate M1/M2 subsets of polarized macrophages, which play juxtaposing roles in the inflammatory cascade. ^68^Ga-DOTATATE targets the somatostatin receptor subtype-2 but is limited by its inability to distinguish beneficial from harmful inflammation.^42^

Alternatively, molecular MRI using MPO-Gd provides selective and direct information on detrimental plaque inflammation induced by extracellular MPO. It detects pathologic plaque activity with high sensitivity and resolution and can be coupled with established multi-contrast MRI sequences, which characterize high-risk plaque components,^8,43^ enhancing our understanding of plaque vulnerability and providing a more accurate risk stratification. The present study demonstrates this synergy and further strengthens the potential of imaging MPO activity to identify thrombosis-prone, culprit lesions.

Molecular imaging of MPO activity using MPO-Gd or its DOTA-MPO analogue with superior safety and imaging profile^44^ appears to be realistic candidates for translational use to aid the clinical detection of vulnerable plaques for treatment escalation, intervention, and surveillance. These agents have good biocompatibility^45^ as their main chemical components naturally occur in humans (5-hydroxytryptamine) or are already used in clinical imaging (Gd-DTPA or Gd-DOTA). The imaging window (30–90 min post-injection) and injected dose (0.1 mmol/kg, comparable to that used in patients) make this approach suitable and practical for clinical application. Moreover, our *in vivo* animal experiments were conducted on a clinical scanner with vessel wall imaging protocols and quantitative T_1_ mapping sequences used in patients. Moreover, new accelerated image acquisition and reconstruction methods further minimize the barrier for clinical use of MRI for plaque imaging by reducing scan time and costs. As gadolinium agents have higher *r1* relaxivity at lower field strengths resulting in a stronger signal, recent commercialization of clinical low-field MRI scanners (0.55 T) may further facilitate translation of molecular MRI of MPO activity by reducing the injected dose.

Another avenue to expedite clinical translation is through the utilization of a PET-based probe for the detection of MPO activity. PET imaging probes face a less stringent pathway to human use given the low concentrations of radiolabelled tracer administered, their short half-life, and the comparative ease of probe development to gadolinium-based agents. A fluorine-18-based PET imaging radioprobe (^18^F-MAPP) has been validated for the selective detection of MPO activity^46^ and is currently undergoing approval for human use in the USA. Nevertheless, ^18^F-MAPP is cell permeable and therefore measures total MPO activity rather than extracellular MPO activity unlike the MRI probe used in this study. As such, work on the development of a PET-based probe specific for the detection of extracellular and hence pathological MPO activity continues.

### Study limitations

In the rabbit model, plaque disruption does not happen spontaneously but required exogenous triggers, although spontaneous atherothrombosis is very scarce in animal models of atherosclerosis.^47^ The rabbit model also lacks plaque calcification and IPH, i.e. features common in human plaques and that associate with lesion instability. Due to the nature of human tissue collection, it was not possible to assess whether MPO activity predicts plaque rupture in humans, though the rabbit data were specifically utilized to support this hypothesis. Finally, it was not possible to assess MPO activity in human tissue *in vivo*. As a result, the assessment of the probe in this study was limited to *ex vivo* soaking, probe activation, and washing of CEA specimens. Finally, despite our study being observational, the preclinical data strongly support the predictive value of molecular imaging of plaque MPO activity in detecting atherothrombosis. The clinical data are complementary and strengthen the case for future clinical translation.

## Conclusion

*In vivo* imaging of MPO activity using an MPO-Gd molecular probe predicts future atherothrombosis in a preclinical model, and MPO-Gd is retained selectively in ruptured human atherosclerotic plaque. These results highlight the specific role of MPO activity and its potential application as a molecular target in the detection of high-risk atherosclerotic plaque. Future *in vivo* human trials confirming the novel findings of this study are warranted.

## Supplementary Material

qyae004_Supplementary_Data

## Data Availability

Raw data involved in this study are available from the corresponding authors upon reasonable request.

## Lead author biography

James Nadel is a cardiologist and cardiovascular researcher from Australia with interest in non-invasive cardiovascular imaging and its application in atherosclerosis. His PhD focused on exploring the role of myeloperoxidase in vulnerable plaque and its potential applications as an imaging biomarker. He has presented and published research on this topic internationally and was the recipient of the European Association of Cardiovascular Imaging Young Investigator Award in 2023.

Dr Xiaoying Wang is a research associate at King’s College London, specializing in medical imaging and drug testing for cardiovascular disease. Her current focus is on innovative MRI and treatment strategies for atherosclerotic plaques by targeting myeloperoxidase activity. Dr Wang earned her PhD from Clemson University, USA, where her research centred on the application of nanotechnology to develop targeted CT contrast for risk stratification and a drug delivery system for the treatment of abdominal aortic aneurysms. Her research has resulted in numerous publications and presentations at international conferences, underscoring her dedication to advancing cardiovascular health through cutting-edge techniques.
