# Supplementary material for: Molecular magnetic resonance imaging of myeloperoxidase activity identifies culprit lesions and predicts future atherothrombosis
Source: Eur Heart J Imaging Methods Pract. 2024 Jan 24;2(1):qyae004. doi: 10.1093/ehjimp/qyae004 (PMC10870993; doi:10.1093/ehjimp/qyae004)
Supplement: qyae004_Supplementary_Data [file qyae004_Supplementary_Data.docx]

**Supplemental Material**

***Rabbit Model of Atherothrombosis***

Atherosclerosis was induced in 8–11-weeks-old male, New Zealand white rabbits (n = 12, ~2.5 kg), as described previously^1^ (**Figure S1A**). Rabbits were fed with a chow diet supplemented with 1% (wt/wt) cholesterol for 8 weeks to induce hypercholesterolaemia. Anaesthesia was induced by intramuscular ketamine (Ketamidor, Chanelle Pharma, Ireland, 35 mg/kg) and xylazine (Rompun, Dechra, UK, 5 mg/kg) and maintained by inhalation of 2-4% isoflurane. After 2 weeks of 1% cholesterol diet feeding, aortic endothelial denudation was induced under general anaesthesia using a nominally inflated 3F Fogarty catheter (12TLW403F, Edward Lifesciences, USA) advanced through a 4F sheath into the infrarenal aorta through a right femoral artery cutdown. Endothelial denudation was achieved by three sequential manual pullbacks over approximately 90-100 mm. The artery was then ligated, and muscular and skin closure with 3-0 vicryl sutures followed. After 8 weeks of 1% cholesterol feeding, rabbits were placed on normal chow for 4 weeks before triggering atherothrombosis by intraperitoneal administration of Russell’s Viper Venom (0.15 mg/kg, Enzyme Research, UK) followed by an intravenous injection of histamine (0.02 mg/kg, MP Biomedicals, USA) 30 min later. The triggering process lasted 8 h with two triggers performed 4 h apart. Age- and gender-matched rabbits (n=3) were used as controls to measure the aortic wall area. Rabbits were euthanised after the post-trigger MRI session with an intravenous injection of 100 mg pentobarbitone/kg (Pentoject, 20% (wt/vol), Animalcare, UK). The abdominal aorta was marked from the left renal artery to the iliac bifurcation measured *in situ* prior to being dissected, then stretched to its physiological length, and pinned to a corkboard. All samples were stored at -80 °C until further analysis. All animal use was in accordance with local rules and approved by King’s College London, Animal Welfare and Ethical Review Board, London, UK, project licence PP8261525, following the regulations and guidance issued under the Animals (Scientific Procedures) Act (1986).

***In Vivo Molecular MRI of MPO Activity in Rabbits***

*In vivo* MR images of the abdominal aorta were acquired using a 3.0 Tesla Philips Achieva clinical scanner (Philips Healthcare, Best, Netherlands) equipped with a clinical gradient system (30 mT/m, 200 mT/m per ms). Rabbits were anaesthetised and positioned supine during the scan. Data acquisition was performed with a 32-channel cardiac coil and gated with a simulated electrocardiogram. Rabbits were scanned three times (**Figure S1A**): once at 8 weeks (at the end of the cholesterol feeding period), and twice at 12 weeks pre- and post-trigger of atherothrombosis. For the first two scans MPO-Gd was reconstituted in 5 mL phosphate-buffered saline pH 7.4 (PBS) containing 2% (vol/vol) dimethyl sulfoxide, sonicated, and heated to 60 °C until dissolved, before being injected intravenously (0.1 mmol/kg) 45 min prior to data acquisition as described previously and detailed below.^2^ For the third scan, carried out 4 h after the second trigger event, T1-weighted black blood (T1BB) images were acquired at 0, 10, and 20 min without contrast agent to dynamically monitor developing thrombi. Coronal phase-contrast angiograms were acquired for visualisation of the aorta, renal branches, and iliac bifurcation with the following parameters: FOV = 300 x 150 x 15 mm, matrix = 200 x 100, resolution = 1.5 x 1.5 mm, slices = 15, slice thickness =1 mm, TR/TE = 20/3 ms, flip angle = 15 °, and phase-contrast velocity = 150 cm/s. The maximum intensity projection images with full vision of the abdominal aorta from the left renal branch to iliac bifurcation were used to plan the subsequent images.

Electrocardiographically (ECG) triggered transverse multi-slice 2D zoom (reduced FOV) double inversion recovery T1BB images were acquired with the following parameters: FOV = 70 x 19 x 100 mm, matrix = 280 x 69, resolution = 0.25 x 0.25 mm, slices = 20, slice thickness = 5 mm; TR = two heartbeats, TE = 11 ms, flip angle = 15 °, and BB inversion delay = 350 ms. A two-dimensional Look-Locker sequence was used to determine the optimal inversion time for blood signal nulling before and after administration of the MPO-Gd with the following parameters: FOV = 150 x 150 mm, matrix =152 x 150, resolution =1 x 1 mm, slice thickness = 10 mm; and TR/TE = 10/4.5 ms. T1-weighted non–ECG gated 3D inversion-recovery (T1w-IR) gradient-echo transverse images were acquired before and 1 h after administration of MPO-Gd with the following parameters: FOV = 110 x 85 x 99 mm, matrix = 440 x 340, resolution = 0.25 x 0.25 mm, slices = 33, slice thickness = 3 mm, TR/TE = 7.1/2.8 ms, time between subsequent inversion-recovery pulses = 1,000 ms, and flip angle = 30 °. T1 mapping was performed 1.5 h after administration of MPO-Gd using a 3D Look-Locker-based inversion recovery gradient echo sequence with a segmented k-space acquisition to allow acquisition of T1 maps at a higher spatial resolution, which is crucial for vessel wall mapping. This 3D gradient-echo sequence commenced with a single non-selective inversion pulse, followed by the acquisition of 30 inversion recovery images with inversion times ranging from 10 - 8,190 ms. T1 mapping parameters were as follows: FOV = 86 x 65 x 102 mm, matrix = 214 x 164 mm, resolution = 0.4 x 0.4 mm, slice thickness = 6 mm, flip angle = 10 °, TR/TE = 5.5/2.9 ms and slices = 17. The T1 maps were reconstructed off-line, using SENSE ^3^ and coil maps estimated by ESPIRiT ^4^ to reconstruct the intermediate T1 weighted images. A dictionary (list of magnetization signal evolutions) was computed using the Look-Locker model ^5^ for tissues with T1 in the range of 100 – 3,000 ms, with a step of 1 ms. Inner-product between the dictionary and each pixel in the T1 weighted images were evaluated to identify the best match in the dictionary and reveal the corresponding T1 value, as commonly used in magnetic resonance fingerprinting.^6^ The R1 maps were generated by transforming the T1 maps in Matlab_R2021b using the function: R1 = 1,000/T1. The R1 maps were then imported into Osirix, and the vessel wall was segmented manually to obtain the R1-values.

***Grouping, Masking and Analysis of Rabbit MR Images***

MR images were analysed on a slice-by-slice basis. In each diseased rabbit, the injured abdominal aorta consisted of lesion-free segments and segments containing plaques with different phenotypes (**Figure S1B-1C**). Lesion-free and plaque-containing segments were distinguished based on the aortic wall area (AWA) determined in age-matched healthy control (n=3) and diseased rabbits (n=12) (**Figure S2**). Firstly, the adventitia and the lumen of the aortic wall were segmented manually using the pre-trigger T1BB images. The AWA was then calculated over 60 MR images as adventitial area minus luminal area. The mean AWA of control rabbits (AWA_healthy_) was used to define a threshold to distinguish lesion-free from plaque-containing segments in diseased rabbits, with AWA_diseased_ ≤ AWA_healthy_ + 2SD considered to represent lesion-free segments and AWA_diseased_ > AWA_healthy_ + 2SD to reflect plaque-containing segments (**Figure S2**). Pre- and post-trigger MR images were then matched using the left renal artery and iliac bifurcation as internal anatomical landmarks. Plaque-containing segments were classified further into stable (defined as plaques resistant to trigger-induced thrombosis) and thrombosis-prone plaques (defined as plaques that developed thrombosis following trigger) based on the absence and presence of a thrombus on native (contrast-free) post-trigger T1BB images, respectively (**Figure S2**). For image masking, the mean signal intensity (SI) of arterial wall segments deemed as lesion-free (SI_lesion-free_) was calculated using T1w-IR MPO-enhanced images. Subsequently, signal intensity > SI_lesion-free_ + 2SD, observed in stable and thrombosis-prone plaque, was considered to represent retention of MPO-Gd due to MPO plaque activity. Before quantitative analysis, all images were reviewed by an experienced observer. Pre-trigger T1BB images were analysed for AWA. To study late MPO-Gd-enhancement (LGE), pre-trigger T1w-IR images and T1 maps were masked and used for calculating the enhanced area and the corresponding aortic tissue-to-muscle contrast ratio and R1-values. Data were plotted for each of the three types of arterial segments described above, *i.e.*, lesion-free, stable plaque-containing and thrombosis-prone plaque-containing.

***Histology of Rabbit Aortic Segments***

Aortic segments (lesion-free, stable plaque and thrombosis-prone plaque; n=3 per type) were selected for histological analysis based on the MR images and gross views indicating the absence/presence of plaque/thrombi (**Figure S1C**). Segments were dissected, paraffin-embedded, transversely sectioned (5 µm), and stained at 56 °C for 20 min with Masson’s Trichrome using Bouin’s solution as a mordant, rinsed with running tap water, and then stained further with Weigert’s iron haematoxylin, Biebrich scarlet/acid fuchsin, and aniline blue (Trichrome Stain (Masson) Kit, Sigma, HT15-1KT) to study the general morphology of the plaque. For the detection of MPO protein, tissue sections were blocked sequentially with 0.3% (vol/vol) hydrogen peroxide (H_2_O_2_; Sigma-Aldrich) for 10 min and 5% (wt/vol) bovine serum albumin (Sigma) for 1 h at room temperature. A monoclonal mouse anti-human MPO antibody (Abcam ab10165; 1:400 dilution) was used at a concentration of 0.1 mg/mL for immunohistochemical detection of MPO protein. Incubation with the primary antibody was carried out overnight at 4 °C, followed by incubation for 1 h at room temperature with a secondary antibody (Dako P0447, goat anti-mouse IgG/horseradish peroxidase conjugate, 1:500 dilution). A DAB substrate kit (Dako) was used to visualise the signal and hematoxylin was used for counterstaining of nuclei.

***Human Ethics***

A single-centre prospective trial recruiting patients scheduled for carotid endarterectomy was performed between February 2020 and February 2022. Where feasible, participants underwent *in vivo* carotid MRI within 7 days prior to their surgery. The clinical study was approved by the Human Research Ethics Committee of St Vincent’s Hospital, Darlinghurst, Australia (2019/PID13059). All participants provided written informed consent.

***In Vivo Carotid MRI In Patients***

*In vivo* MRI was performed as described previously^7^ using a 3.0 Tesla Siemens MAGNETOM scanner (Siemens Healthineers, Erlangen, Germany) using two coils, with a head and neck 20 coil and special purpose 4 coil positioned ipsilateral to the carotid plaque of interest. Following 3-dimentional (3D) gradient echo (GRE) scout scans, transverse 3D time-of-flight (TOF) magnetic resonance angiography (MRA) images were used to visualise the extracranial head and neck vessels with a field-of-view (FOV) = 160 x 160 x 48 mm, matrix = 256 x 256, in-plane resolution = 0.6 x 0.6 mm, slice thickness = 1.0 mm, slice slab = 48 mm, repetition time (TR) = 24 ms, echo time (TE) = 3.47 ms, and flip angle = 20 °. The maximum intensity projection images were used to plan the subsequent scans. 2D fat suppressed quadruple inversion recovery black blood T1 weighted turbo spin echo (T1w-IR) images were acquired with a FOV = 160 x 160 x 32 mm, matrix = 256 x 256, in-plane resolution = 0.6 x 0.6 mm, slice thickness = 2.0 mm, slices = 16, TR = 800 ms, TE =12 ms, flip angle = 160 °. 2D fat suppressed, multi-slice double inversion-recovery T2 weighted turbo spin echo (T2w) scans were acquired with FOV = 160 x 160 x 32 mm, matrix = 256 x 256, in-plane resolution = 0.6 x 0.6 mm, slice thickness = 2.0 mm, slices = 16, TR = 4,800 ms, TE = 47 ms, and flip angle = 160 °. 2D Magnetization-prepared, rapid gradient-echo (MPRAGE) images were acquired with FOV = 160 x 160 x 48 mm, matrix = 256 x 256, in-plane resolution = 0.7 x 0.7 mm, slice thickness = 0.9 mm, slices = 104, TR = 13 ms, TE = 4.0 ms, flip angle = 9 °, and inversion recovery pulse delay = 920 ms. Post-contrast enhanced (CE) T1w images were acquired 5 min following intravenous injection Gadovist^®^ (Bayer, Leverkusen, Germany) (0.1 mmol/kg).

*In vivo* MRI data were analysed on a slice-by-slice basis by two separate readers who provided an MRI-adapted American Heart Association (AHA) plaque grade^8^ using four sequential slices proximal and distal to the carotid bifurcation. The presence of atherosclerotic plaque features including a lipid-rich necrotic core, calcification, intraplaque haemorrhage (IPH), thrombus, and ruptured fibrous cap was assessed using corresponding T1-weighted (T1w), T2w, MPRAGE, angiography and post-contrast enhanced T1w MRI. When interobserver discordance occurred, consensus was obtained by reading sequences together.

***Human Carotid Endarterectomy (CEA) Procurement and Processing***

The workflow for CEA specimen collection is described in **Figure S5**. Specimens (n=30) were collected from patients undergoing CEA for either symptomatic or asymptomatic severely stenotic carotid artery disease. Freshly obtained specimens were purged of blood and then stored at -80 °C for subsequent analyses. Where pre-surgical *in vivo* carotid MRI was performed (n=12), plaques were kept whole for *ex vivo* MRI analysis. Alternatively, whole plaques (n=18) were divided into 3-5 mm sections with sequential sections undergoing *ex vivo* MRI, histological assessment or MPO activity determination, respectively.

***Ex Vivo MRI Protocol for Whole Human Plaque and Tissue Section Scans***

All *ex vivo* imaging experiments were performed using a 9.4T Bruker BioSpec 94/20 Avance III micro-imaging system (Bruker, Ettlingen, Germany) equipped with BGA-12S HP gradients with maximum strength 660 mT/m and slew rate 4,570 Tm/s. Image acquisition used a 23 mm Quadrature Volume Receive/Transmit RF-coil (Bruker, Ettlingen, Germany). MPO-Gd was custom-synthesised by Peptide Synthetics (Peptide Protein Research Ltd, Hampshire, UK) following the synthetic route described previously.^2^ Images were acquired using a 2-sequence protocol, consisting of high resolution T1 anatomical imaging and quantitative T1 mapping. Anatomical imaging was performed using a 2D Fast Spin Echo (RARE) pulse sequence (to match the clinical imaging as closely as possible) at high resolution using the following acquisition parameters: TR = 1,500 ms, TE = 6.3 ms, echoes/excitation = 4, FOV = 15 x 15 mm, matrix = 256 x 256, in-plane resolution = 60 x 60 µm, slice thickness = 0.5 mm, inter-slice gap = 0.2 mm, and number of averages = 24. T1 quantification was performed using a saturation recovery technique optimized for the expected range of T1-values in atherosclerotic plaque, and slice thickness roughly matched to that used in clinical scans. The sequence used a variable TR 2D Fast Spin Echo method with six different saturation recovery times and the following base parameters: TR range = [300, 400, 800, 1,500, 3,000, 5,500 ms), TE = 7 ms, echoes/excitation = 2, FOV = 15 x 15 mm, matrix = 256 x 256, in-plane resolution = 60 x 60 µm, slice thickness = 1 mm, inter-slice gap = 0.4 mm, and slices = 15. The total acquisition time with 2 number of averages was 36 min per specimen.

***Human Sample Set Up and Positioning***

To allow for reproducible positioning and to facilitate precise co-registration of images from different timepoints, a disposable, 2-part sample holder was designed, and 3D printed (**Figure S6**). The first part consisted of a grooved spacer that was glued firmly into a standard 25 mL syringe. The second part, a removable specimen grid, was inserted and held in place by the fixed groove and a syringe plunger. This sliding mechanism allowed for highly reproducible positioning of the grid relative to the imaging window and subsequent image alignment. Tissues were glued on top of the sample grid by applying superglue sparingly to the grid junctions and slowly settling the specimen into the glue droplets. After sliding the specimen-containing sample grid into the holder, the syringe was filled with Perfluorinated Polyether (Fomblin™ 6Y) to reduce image distortions from susceptibility discontinuities (air-tissue interfaces), and then closed using the syringe plunger and matching cap. Finally, the syringe was inserted into the imaging coil and positioned at the scanner isocentre for image acquisition. Imaging protocols used for CEA sections varied from those used for whole CEA specimens. For the analysis of CEA tissue sections, 3-5 mm thick sections were imaged at baseline and at the established timepoints following MPO-Gd activation.

***Human CEA Specimen Preparation for Ex Vivo Molecular MRI***

To assess MPO-activity in CEA specimens *ex vivo*, the specimen was immersed in Gd-MPO formulation followed by washing to remove free floating residues of the tracer from the sample. A similar method has previously been used to investigate MPO-Gd retention in human livers.^9^ The conditions for probe exposure and washing were optimised first for CEA tissue to delineate non-specific from specific retention of the probe. For this, diethylenetriamine pentaacetic acid-gadolinium (DTPA-Gd) was used as a model compound for MPO-Gd. DTPA-Gd lacks the MPO-specific targeting moieties of MPO-Gd, while the Gd ion in MPO-Gd is chelated to DTPA and Gd can be readily quantified using inductively coupled plasma mass spectrometry (ICP-MS). To assess optimum immersion times for full specimen penetration, CEA segments were soaked at 37 °C in 10 mL PBS containing physiological concentrations of glucose (1 mg/mL) and 0.45 mg/mL DTPA-Gd (Magnevist^®^) for increasing periods of time. After soaking, samples were rinsed briefly with fresh PBS and blotted dry. The contrast agent retained in the tissue was then quantified by assessing Gd concentrations in the bulk tissue by ICP-MS, with the amounts of Gd normalised to dry weight of tissue. Based on the ICP-MS data obtained (**Figure S7A**), 1 h soaking was considered sufficient for adequate absorption.

Next, a series of washing experiments were performed to optimise the removal of residual untargeted contrast agent from the tissue samples. For this, CEA tissues were soaked in DTPA-Gd-containing PBS at 37 °C for 1 h and then washed in PBS at 37 °C for the time indicated, with PBS exchanged every 2 h. ICP-MS data showed that after 4-6 h total Gd concentrations largely returned to close to baseline values before Gd-DTPA immersion (**Figure S7B**). Therefore, a washing time of 4-6 h was considered to provide adequate washing out of all untargeted DTPA-Gd, and Gd retention beyond the 4–6 h of washing attributed to specific retention of MPO-Gd due to MPO activity.

The specific retention of MPO-Gd in MRI experiments was then determined using the previously established MRI protocols and soaking and washing conditions. To investigate targeted retention, CEA specimens with histologically confirmed stable or ruptured plaques were chosen. To assess multiple washing periods, MRI was performed at multiple timepoints according to the protocols described above, with a first MRI acquired as baseline at the start of the experiment before any treatment of the specimen. As specific targeting by the MPO-Gd probe requires the presence of active MPO, which in turn requires H_2_O_2_, glucose oxidase was used in conjunction with glucose to form H_2_O_2_ at a steady rate in our specimen immersion solutions, according to the following protocol: First, tissues were submerged for 30 min at 37 °C in PBS containing MPO-Gd (5 mg/mL) and glucose oxidase (1 µg/mL) to allow for penetration of the MPO-Gd probe into the tissue, thus ensuring that the probe has enough time to reach relevant sites before activation. Second, glucose (8 mg/mL) was added to generate H_2_O_2_ inside the soaking solution. Preliminary studies (data not shown) revealed that under these experimental conditions H_2_O_2_ was formed at a linear rate of 0.5 nmol/min for ~30 min, as assessed by xylenol orange oxidation. To allow for activation, the specimens were left in this ‘activating solution’ for 30 min after glucose addition. After Gd-MPO treatment, specimens were washed briefly using PBS and then underwent a second MRI as the first, immediate post-contrast MRI. Specimens then underwent multiple repeats of 2 h ‘washing’ where specimens were submersed in PBS to increasingly wash out any MPO-Gd that was not retained specifically. From previous untargeted retention experiments a total washing period of 6 h was chosen with MRI being performed after every 2 h washing step.

All MR images were acquired according to the protocols described above and analysed using the procedures below. To assess probe retention from MRI, bulk Spin-Lattice relaxation (R1) and relative relaxation change (ΔR1) were measured from MRI T1 relaxation maps as average across ROIs inside histology confirmed stable and unstable plaque. The temporal behaviour of R1 and ΔR1 was then plotted for the assessed washing periods to determine an optimal protocol. Results of these evaluations are shown in **Figure S7C-D**. From these results a period of 4 h following post-contrast imaging was determined appropriate and feasible to differentiate non-activated from activated/retained MPO-Gd in ruptured (n=6) compared with stable plaques (n=8).

***Assessment Protocol for Whole Human CEA Specimens***

The final protocol that emerged from the above development phase consisted of a set of 3 MRI sessions that were interleaved with treatment and washing periods, respectively. The timeline of the protocol was as follows: 1. T_bsl_: Pre-Contrast (baseline) MRI; 2. T_0_: Gd-MPO treatment; 3. Post-contrast MRI; 4. Four hours washing in PBS, 5. MRI 4 h post contrast and washing. Like the development phase study, whole CEA specimens were fixed to a 3D printed sample holder as described above, and then inserted into a 25 mL syringe and submerged in perfluorinated polyether for imaging.

***Ex Vivo MRI Image Analysis of Human CEA Specimens***

Quantitative T1 (R1) values and their relative local T1 recovery were used as surrogate markers to determine *ex vivo* retention of MPO-Gd. The validity of this approach has been established previously in studies using different animal models and phantoms.^2,10^. Quantitative T1 maps were calculated from the saturation recovery MRI by pixelwise fitting of an exponential saturation recovery model to the image signals measured across the images at different repetition times. The resulting raw T1 maps were then masked from the background using Otsu thresholding. T1 maps from baseline and subsequent imaging timepoints were processed further to calculate percentage recovery maps for each specimen. Images were co-registered using a landmark co-registration method to eliminate residual misalignment before calculating maps of relative T1 recovery. From these, percent T1 recovery maps were quantified by pixelwise algebra using the equation ΔT1(t_x_) = 100 x T1(t_x_)/T1_bsl_, where T1(t_x_) is the pixel value of the T1 map at timepoint t_x_ after washing, and T1_bsl_ is the respective pixel value of the baseline T1 map. The relative maps were then used to quantify retention in the development phase using CEA sections with histological correlation, as well as in the final study phase employing whole plaque.

In the development phase, ΔT1(t_x_) maps were compared with histological analyses of the adjacent tissue to establish appropriate schedules for treatment and washing. In the actual study, the results from the developmental phase were used to infer location of vulnerable plaque from these maps. The *ex vivo* data of whole plaques were then correlated with pre-surgical *in vivo* carotid MRI using carotid bifurcation as a reference landmark. MPO-Gd retention was quantified from the *ex vivo* images every 2 mm, proximal and distal to the bifurcation and correlated with plaque assessment from clinical MRI as well as by AHA grading.

***Histology of Human CEA Samples***

Tissues were formalin fixed, decalcified (3.5% nitric acid containing 50 mg urea/L), paraffin-embedded and cross sectioned (5 µm). Each tissue section was graded using the AHA histological classification of atherosclerosis.^11^ To aid plaque classification, the following stains were used: haematoxylin and eosin, picrosirius red for visualising intact versus ruptured fibrous caps, Perls’ Prussian blue stain for haemosiderin deposition indicative of prior haemorrhage or thrombosis, and Movat’s pentachrome to delineate plaque constituents including lipid pool, immune cells, arterial wall, and fibrin. Additionally, immunohistochemistry for MPO (polyclonal rabbit anti human, Dako A0398; dilution 1:3,000) and CD68 (anti-CD68 [KP-1] primary antibody, Roche-Ventana 790-2931) was performed to compare regions of probe retention with sites of MPO protein expression and macrophages.

***Plaque MPO Activity Determination for Rabbit and Human Samples***

For human CEA samples, tissue sections adjacent to histologically analysed samples were homogenised. For rabbit tissue, aortic segments that were lesion-free (n=6), containing stable plaque (n=10) or thrombosis-prone plaque (n=5) were selected from different rabbits based on MR images and gross views. Visible thrombi were removed from the tissue prior to MPO activity determination. Human and rabbit samples were prepared and analysed for MPO activity as described previously, using the validated method of the MPO-specific chlorination of the synthetic compound hydroethidine to 2-chloroethidium (2-Cl-E^+^).^12,13^ Briefly, samples were homogenised on ice in homogenising buffer (PBS containing 50 µM diethylenetriaminepentaacetic acid and 1x Roche complete™ protease inhibitor) using a micro tissue glass grinder (Wheaton, USA). Homogenates were centrifuged (6,000 x g for 1 min at 4 °C for CEA and 800 x g for 5 min at 4 °C for rabbit samples). The resulting supernate was collected and its protein content determined by the bicinchoninic acid assay. Using homogenising buffer, samples were then diluted to 10 µg (for CEA) and 100 µg protein (for rabbit arteries) in a volume of 80 µL and kept on ice. To each of these standardised samples, 5 µL of each of the following ice-cold reagents were added in order and on ice: Trolox^®^ (20 mM, dissolved in ethanol), glucose (20 mg/mL homogenising buffer), hydroethidine dissolved in N_2_-sparged ethanol (1 mM for CEA and 2 mM for rabbit samples), and glucose oxidase (40 µg/mL homogenising buffer). Regents were mixed vigorously for 5 s, incubated for 30 min in the dark at 37 °C using a thermal mixer set at 600 rpm, and then immediately transferred onto ice. To each of the reactions, 5 µL internal standard (*d*_5_-2-Cl-E^+^, 3 µM in ethanol) was added. To halt the reaction and extract the internal standard and the MPO-specific product, 2-chloroethidium (2-Cl-E^+^), 20 µL of the reaction mixture were added to 80 µL N_2_-sparged, ice-cold ethanol, and the precipitate kept on ice for 10 min in the dark. Extracts were then centrifuged at 16,000 x g for 15 min at 4 °C and the resulting supernates subjected to LC-MS/MS analysis of *d*_5_-2-Cl-E^+^ and 2-Cl-E^+^. The recovery of *d*_5_-2-Cl-E^+^ was used to correct the amount of 2-Cl-E^+^ in the samples. MPO activity was then expressed as pmol 2-Cl-E^+^ per mg protein (mgp).

***LC-MS/MS Analysis***

Liquid chromatography tandem mass spectrometry (LC-MS/MS) analyses were performed using a Shimadzu Nextra UHPLC-40 connected to an 8050 triple quadrupole mass spectrometer (Shimadzu Corporation) as described.^12-14^ Briefly, the following parameters were used: solvent A, 0.1% (vol/vol) formic acid in water; solvent B, 0.1% (vol/vol) formic acid in acetonitrile; injection volume, 2 and 10 µL for human and rabbit samples respectively; oven temperature, 25 **°**C; flow rate, 0.2 mL/min; nebulising gas flow, 2 L/min; heating gas flow; 4 L/min; interface temperature, 400 **°**C; DL temperature, 150 **°**C; heat block temperature, 500 **°**C; drying gas flow, 4 L/min. Reaction extracts were separated using a reversed-phase column (Synergy Polar C_18_ reverse phase, 250 x 2.1 mm, 4 µm). Multiple reaction monitoring (MRM) transitions were optimised for precursor ion *m/z*, product ion *m/z*, Q1 Pre Bias, collision energy, and Q3 Pre Bias prior to analyses, as described previously.^12-14^ Data analysis was performed using the LabSolutions Insight Explore software (Shimadzu Corporation).

***Statistical Analyses***

All analyses were performed using GraphPad Prism statistical software (v. 9.0.1). Quantitative data were presented as box and whisker plots. Data was assessed for normality using the Shapiro-Wilk test. For normally distributed data, univariate parametric statistical analyses were performed using a Student’s *t*-test for two group comparisons or a one-way ANOVA for multiple groups with Tukey’s test for post hoc comparisons between groups. For non-normally distributed data, a Mann-Whitney U test was used for two group comparisons and a Kruskal-Wallis test followed by Dunn’s post hoc test for multiple group comparisons. For the rabbit study, a receiver operating characteristic (ROC) curve and the corresponding area under the curve (AUC) were obtained to assess the value of each MRI-derived measurement (aortic wall area, late MPO-Gd-enhanced area, tissue-to-muscle contrast ratio, and R1 relaxation rate) to predict trigger-induced thrombosis. Statistical significance was defined at a two-tailed *P*-value of <0.05.

**References**

1. Phinikaridou A, Hallock KJ, Qiao Y, Hamilton JA. A robust rabbit model of human atherosclerosis and atherothrombosis. J Lipid Res 2009;50:787-797.

2. Rodriguez E, Nilges M, Weissleder R, Chen JW. Activatable magnetic resonance imaging agents for myeloperoxidase sensing: mechanism of activation, stability, and toxicity. J Am Chem Soc 2010;132:168-177.

3. Pruessmann KP, Weiger M, Scheidegger MB, Boesiger P. SENSE: sensitivity encoding for fast MRI. Magn Reson Med 1999;42:952-962.

4. Uecker M, Lai P, Murphy MJ, Virtue P, Elad M, Pauly JM, et al. ESPIRiT--an eigenvalue approach to autocalibrating parallel MRI: where SENSE meets GRAPPA. Magn Reson Med 2014;71:990-1001.

5. Look DC, Locker DR. Time saving in measurement of NMR and EPR relaxation times. Rev Sci Instrum. 1970;41:250-251.

6. Ma D, Gulani V, Seiberlich N, Liu K, Sunshine JL, Duerk JL, et al. Magnetic resonance fingerprinting. Nature 2013;495:187-192.

7. Sun J, Zhao XQ, Balu N, Hippe DS, Hatsukami TS, Isquith DA, et al. Carotid magnetic resonance imaging for monitoring atherosclerotic plaque progression: a multicenter reproducibility study. Int J Cardiovasc Imaging 2015;31:95-103.

8. Koops A, Ittrich H, Petri S, Priest A, Stork A, Lockemann U, et al. Multicontrast-weighted magnetic resonance imaging of atherosclerotic plaques at 3.0 and 1.5 Tesla: ex-vivo comparison with histopathologic correlation. Eur Radiol 2007;17:279-286.

9. Pulli B, Wojtkiewicz G, Iwamoto Y, Ali M, Zeller MW, Bure L, et al. Molecular MR imaging of myeloperoxidase distinguishes steatosis from steatohepatitis in nonalcoholic fatty liver disease. Radiology 2017;284:390-400.

10. Rashid I, Maghzal GJ, Chen YC, Cheng D, Talib J, Newington D, et al. Myeloperoxidase is a potential molecular imaging and therapeutic target for the identification and stabilization of high-risk atherosclerotic plaque. Eur Heart J 2018;39:3301-3310.

11. Stary HC, Chandler AB, Dinsmore RE, Fuster V, Glagov S, Insull WJ, et al. A definition of advanced types of atherosclerotic lesions and a histological classification of atherosclerosis. A report from the Committee on Vascular Lesions of the Council on Arteriosclerosis, American Heart Association. Arterioscler Thromb Vasc Biol 1995;15:1512-1531.

12. Maghzal GJ, Cergol KM, Shengule SR, Suarna C, Newington D, Kettle AJ, et al. Assessment of myeloperoxidase activity by the conversion of hydroethidine to 2-chloroethidium. J Biol Chem 2014;259:5580-5595.

13. Talib J, Maghzal GJ, Cheng D, Stocker R. Detailed protocol to assess in vivo and ex vivo myeloperoxidase activity in mouse models of vascular inflammation and disease using hydroethidine. Free Radic Biol Med 2016;97:124-135.

14. Vigder N, Suarna C, Corcilius L, Nadel J, Chen W, Payne RJ, et al. An improved method for the detection of myeloperoxidase chlorinating activity in biological systems using the redox probe hydroethidine. Free Radic Biol Med 2023;195:23-35.

**Table S1**. Quantitative analysis of rabbit *in vivo* MRI data at weeks 8 and 12.

|  | **Aortic wall area (mm^2^**) | **Late MPO-Gd enhanced area (mm^2^**) | **Tissue-to-muscle contrast ratio** | **R1, s^-1^** |
| --- | --- | --- | --- | --- |
| **Week 8** | | | | |
| Lesion-free | 4.50 ± 0.94 | 0.16 ± 0.37 | 1.77 ± 1.42 | 1.49 ± 0.17 |
| Stable plaque | 5.58 ± 1.23*** | 1.15 ± 1.35*** | 2.67 ± 2.63* | 1.57 ± 0.20 |
| Thrombosis-prone plaque | 5.70 ± 0.96*** | 1.33 ± 1.50*** | 9.27 ± 8.85^†^ | 2.17 ± 0.37^†^ |
| **Week 12** | | | | |
| Lesion-free | 4.13 ± 0.52 | 0.11 ± 0.40 | 2.65 ± 1.44 | 1.58 ± 0.15 |
| Stable plaque | 5.95 ± 1.18*** | 1.32 ± 1.54*** | 3.21 ± 1.96 | 1.64 ± 0.23 |
| Thrombosis-prone plaque | 5.85 ± 1.06*** | 1.09 ± 1.42*** | 9.37 ± 6.60^†^ | 2.23 ± 0.24^†^ |

Significantly different (**P*<0.05, ****P*<0.0001) from corresponding lesion-free value; ^†^significantly different from corresponding stable plaque value (*P*<0.0001).

**Table S2. ROC Analysis for Imaging Indexes Predicting Trigger-Induced Atherothrombosis in Rabbits**

|  | **Aortic wall area (mm^2^**) | **Late MPO-Gd enhanced area (mm^2^**) | **Tissue-to-Muscle Contrast Ratio** | **R1 (s^-1^)** |
| --- | --- | --- | --- | --- |
| **Week 8** | | | | |
| Area Under Curve | 0.66 | 0.59 | 0.88 | 0.96 |
| 95% CI | 0.57-0.76 | 0.47-0.72 | 0.82-0.94 | 0.93-0.98 |
| p-value | 0.009 | 0.142 | <0.0001 | <0.0001 |
| Cut-off | >5.69 mm^2^ | >0.04 mm^2^ | >2.83 | >1.77 |
| Sensitivity, % | 54.2 | 66.7 | 87.5 | 100.0 |
| Specificity, % | 71.7 | 40.1 | 75.9 | 86.1 |
| **Week 12** | | | | |
| Area Under Curve | 0.69 | 0.52 | 0.91 | 0.97 |
| 95% CI | 0.61-0.77 | 0.40-0.64 | 0.86-0.96 | 0.94-0.99 |
| p-value | 0.003 | 0.796 | <0.0001 | <0.0001 |
| Cut-off | >5.40 mm^2^ | >0.04 mm^2^ | >3.69 | >1.82 |
| Sensitivity, % | 62.5 | 58.3 | 91.7 | 100.0 |
| Specificity, % | 63.2 | 43.4 | 77.4 | 83.9 |

**Table S3. Cohort characteristics**

| **Demographics** | **(n = 30)** |
| --- | --- |
| **Age**, *y* | 69 ± 7.3 |
| **Sex**, *n* (%) |  |
| Male | 22 (73) |
| Female | 8 (27) |
| **Ethnicity**, *n* (%) |  |
| Caucasian | 28 (93) |
| Asian | 2 (7) |
| **Body Mass Index** | 27 ± 7.1 |
| **Comorbidities**, *n* (%) |  |
| Hypertension | 23 (77) |
| Hypercholesterolemia | 23 (77) |
| Diabetes | 10 (33) |
| Active Smoker | 6 (20) |
| **Pharmacotherapy** |  |
| Statin, *n* (%) | 24 (80) |
| Antiplatelet, *n* (%) | 28 (93) |
| Aspirin | 24 (80) |
| Clopidogrel | 8 (27) |
| Anticoagulant, *n* (%) | 8 (27) |
| Heparin | 2 (7) |
| LMWH | 1 (3) |
| DOAC | 4 (13) |
| Warfarin | 1 (3) |
| **Biochemistry** |  |
| Total cholesterol, mmol/L | 4.1 ± 1.5 |
| LDL, mmol/L | 2.1 ± 1.1 |
| HDL, mmol/L | 1.1 ± 0.8 |
| Triglycerides, mmol/L | 1.2 ± 0.7 |
| hsCRP, mg/L | 5.0 ± 4.3 |
| Lipoprotein-a, mg/L | 429 ± 410 |
| Creatinine, µmol/L | 82 ± 33 |
| **Surgical site & indication**, *n* (%) |  |
| Right CEA | 16 (53) |
| Left CEA | 14 (47) |
| Neurological symptoms | 12 (40) |
| Ipsilateral stroke on neuroimaging | 9 (30) |

Abbreviations: CEA, carotid endarterectomy, hsCRP, high-sensitivity C-reactive protein; HDL, high-density lipoprotein; DOAC, direct acting oral anticoagulant; LDL, low-density lipoprotein; LMWH, low molecular weight heparin; n, number; y, year.

**Table S4. *In vivo* MRI for Patient**

| ***In vivo* carotid MRI** |  |
| --- | --- |
| **Analysis**, *n* (%) |  |
| Scans performed | 12/30 (40%) |
| Total slices analysed | 101 |
| Average slices analysed per patient | 8.4 |
| **MRI-based AHA grade**, *n* (%) |  |
| III | 24 (24) |
| IV | 17 (17) |
| V | 43 (43) |
| VI | 17 (17) |
| **Plaque features** |  |
| Luminal stenosis, % |  |
| NASCET criteria | 77±4.0 |
| ESCT criteria | 82±5.4 |
| Total plaque volume, cm^3^ | 0.6±0.8 |
| Total lipid volume, cm^3^ | 0.08±0.1 |
| Total lipid : total plaque volume | 0.13±0.1 |
| Fibrous cap thinning/loss, *n* (%) | 4 (33) |
| Intraplaque hemorrhage, *n* (%) | 4 (33) |
| Thrombus, *n* (%) | 2 (17) |
| **Clinical disposition**, *n* (%) |  |
| Neurological symptoms | 5 (42) |
| Ipsilateral stroke on neuroimaging | 6 (50) |

Abbreviations: ESCT, European Carotid Surgery Trial; NASCET, North American Symptomatic Carotid Endarterectomy Trial; n; number.

**Supplemental Figures**

**
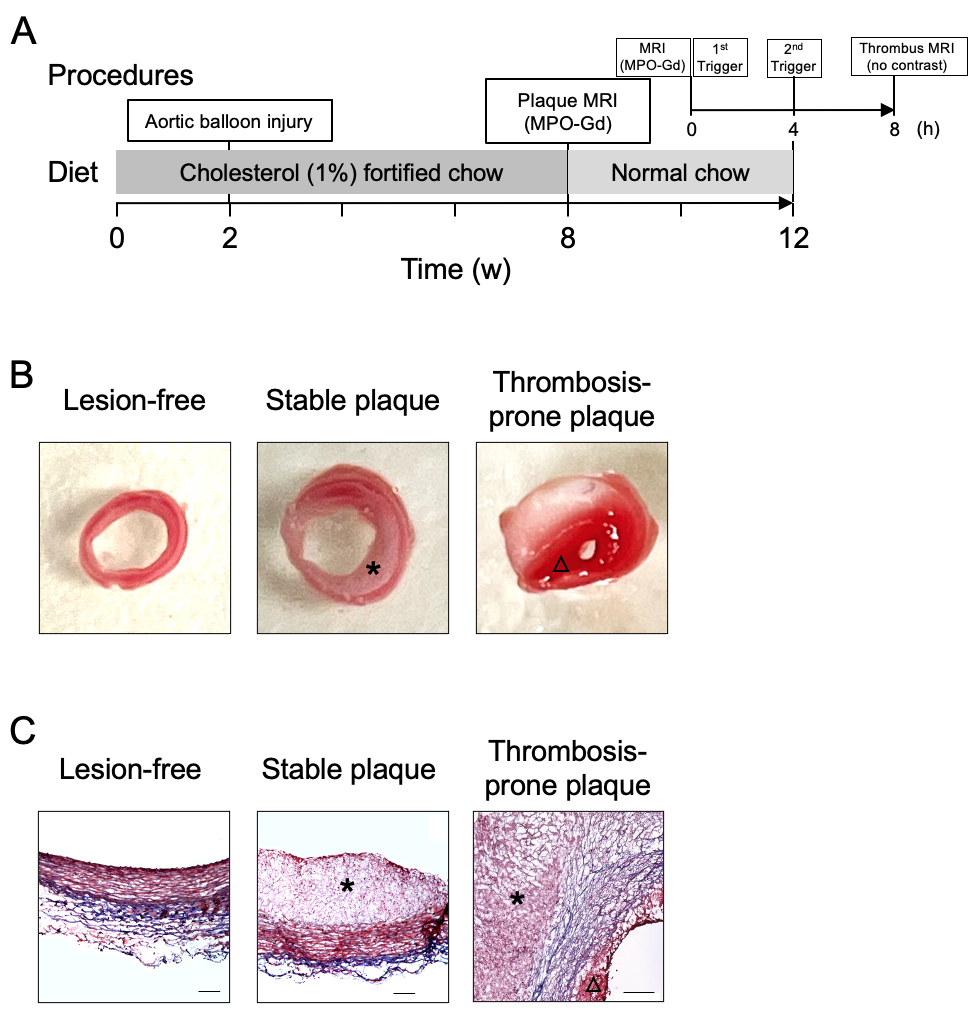
**

**Figure S1**. **Rabbit atherothrombosis study design and model characteristics.** (**A)** Study design. The atherothrombosis model uses male New Zealand White rabbits fed 1% cholesterol-fortified chow diet for 8 weeks, followed by 4 weeks normal chow diet. Endothelial denudation is induced after 2 weeks cholesterol-fortified diet by aortic balloon injury. Thrombosis is triggered pharmacologically after 12 weeks intervention by intraperitoneal injection of Russell’s Viper Venom (0.15 mg/kg) followed by intravenous injection of histamine (0.02 mg/kg) 30 min later, carried out twice, 4 h apart. Molecular MRI using MPO-Gd is performed twice: at week 8 and immediately prior to the 1^st^ trigger at week 12. Non-contrast MRI is carried out 4 h after the 2^nd^ trigger. **(B and C)** Gross view, and Masson’s trichrome staining of three types of aortic segments: lesion-free, containing stable plaque, and containing thrombosis-prone plaque. * and Δ indicate plaque and thrombus, respectively. Scale bars are 200 μm.


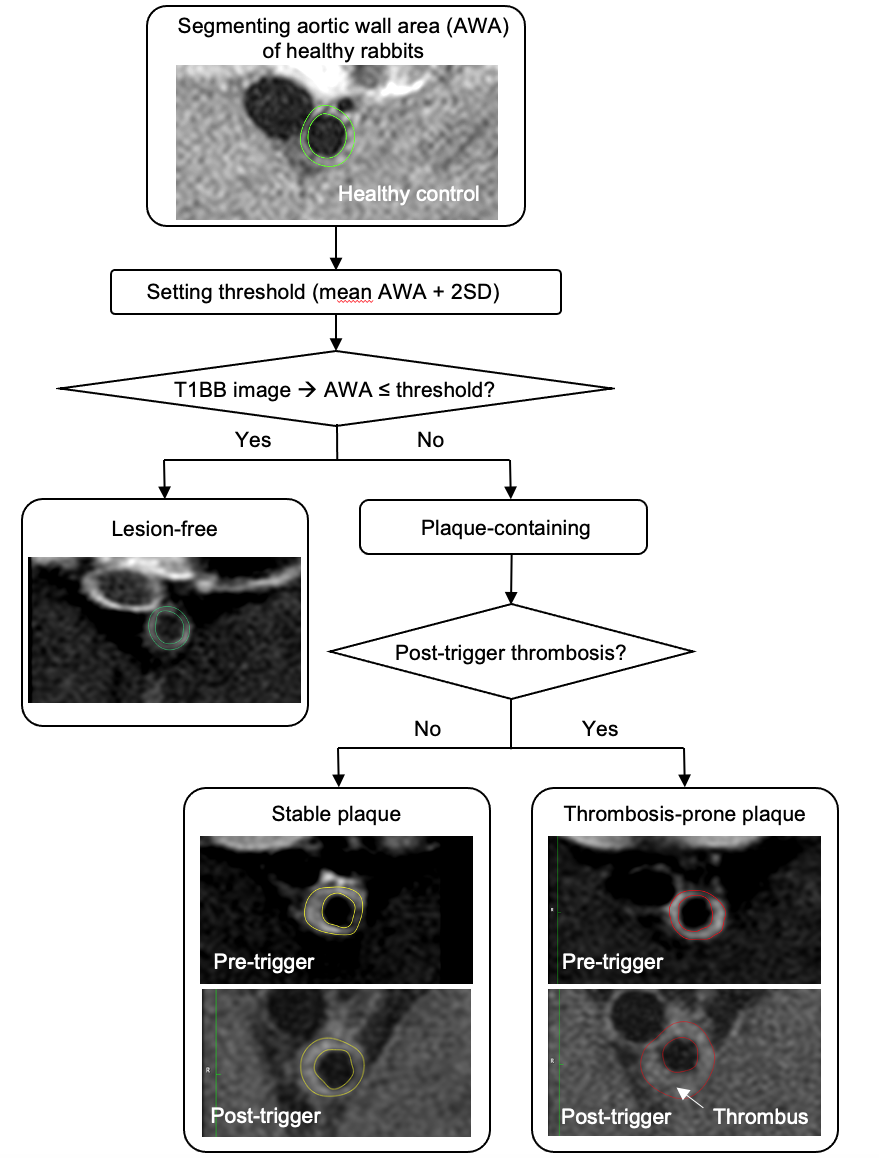


**Figure S2**. **MRI-based classification of aortic segments.** First, an aortic wall area (AWA) threshold was established by segmenting the AWA of three age-matched healthy control rabbits using T1BB images. In diseased rabbits, slices of aortic segments with an AWA ≤ mean AWA + 2 standard deviations (2SD) were classified as lesion-free, whereas aortic slices with AWA > mean AWA + 2SD were classified as plaque-containing segments. Subsequently, using T1BB images acquired after the 2^nd^ pharmacological trigger the plaque-containing segments were divided further into stable plaques (no thrombosis) and thrombosis-prone plaque (with thrombus).


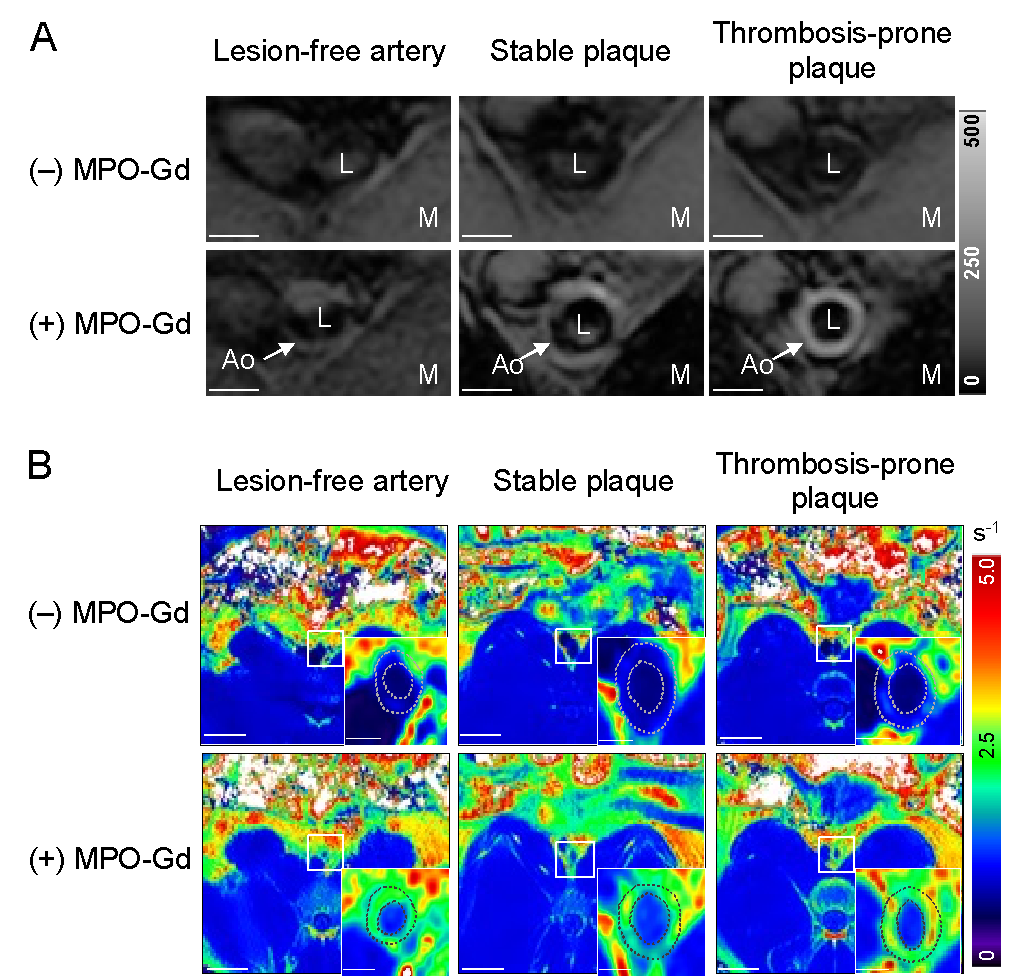


**Figure S3**. *In vivo* pre-trigger MR images of lesion-free abdominal aortic segments and segments containing stable or thrombosis-prone plaque in rabbits 8 weeks after commencement of cholesterol-fortified diet. (**A**) T1w-IR images before (–) and 1 h after (+) MPO-Gd injection. Scale bar, 2 mm. (**B)** R1 maps before (–) and 1.5 h after (+) MPO-Gd injection. Compared with stable plaque, thrombosis-prone plaques showed visually higher late gadolinium enhancement (A) and R1-values (B) after injection of MPO-Gd. Scale bars are 20 and 2 mm for main panels and insets, respectively. Ao, aortic wall; L, aortic lumen; M, paraspinal muscle.


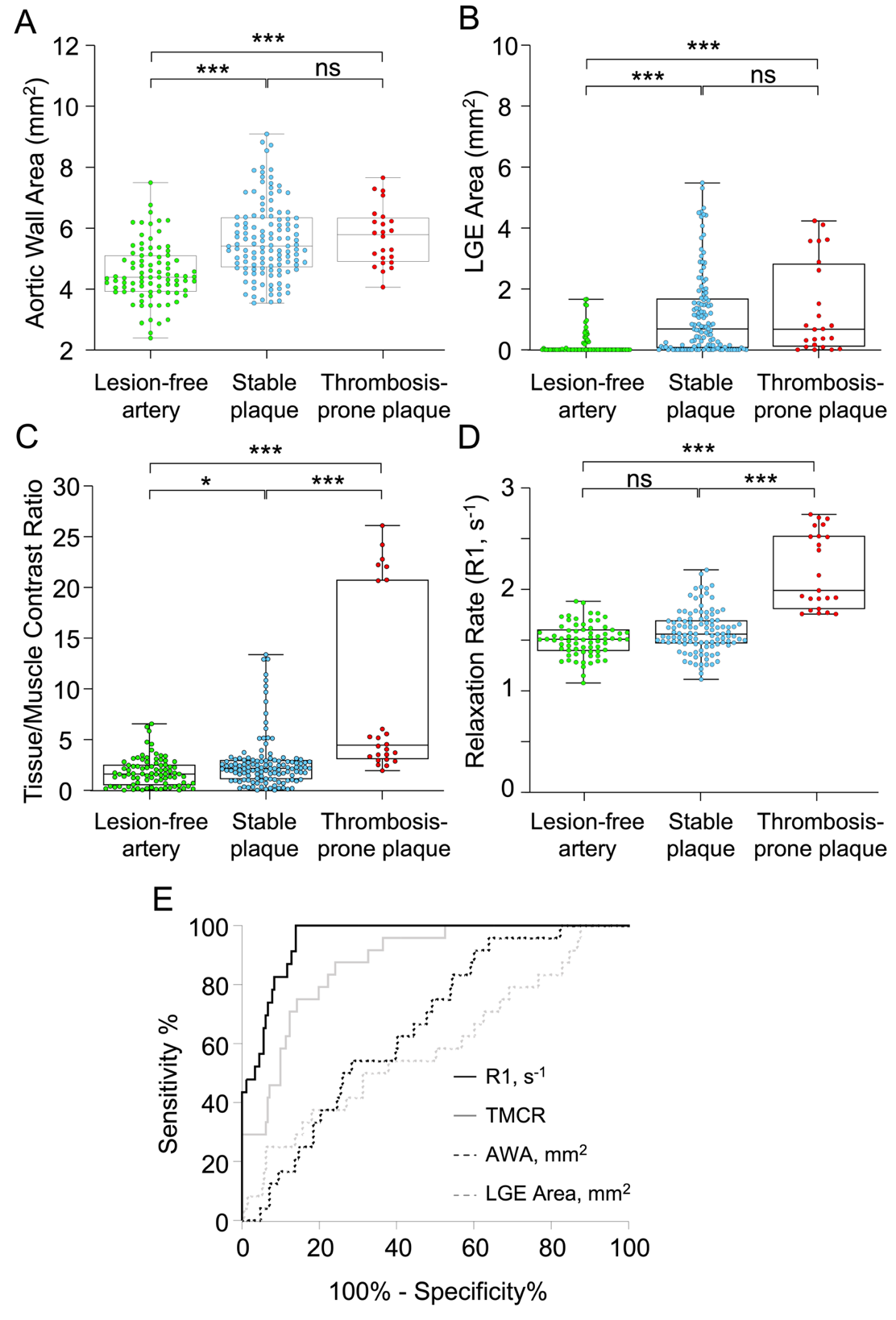


**Figure S4**. Quantitative analysis of MPO-Gd enhanced *in vivo* pre-trigger MR images of lesion-free segments and segments containing stable or thrombosis-prone plaque in rabbits 8 weeks after induction of atherosclerosis. (**A)** Aortic wall area (AWA) segmented from T1BB images. **(B and C)** Late MPO-Gd-enhanced area and tissue-to-muscle contrast ratio (TMCR) based on T1w-IR images 1 h after MPO-Gd administration. (**D)** R1 relaxation rate 1.5 h after MPO-Gd administration. (**E)** ROC curves of the MRI metrics in predicting trigger-induced thrombosis. **P*<0.05, ****P*<0.001.

**
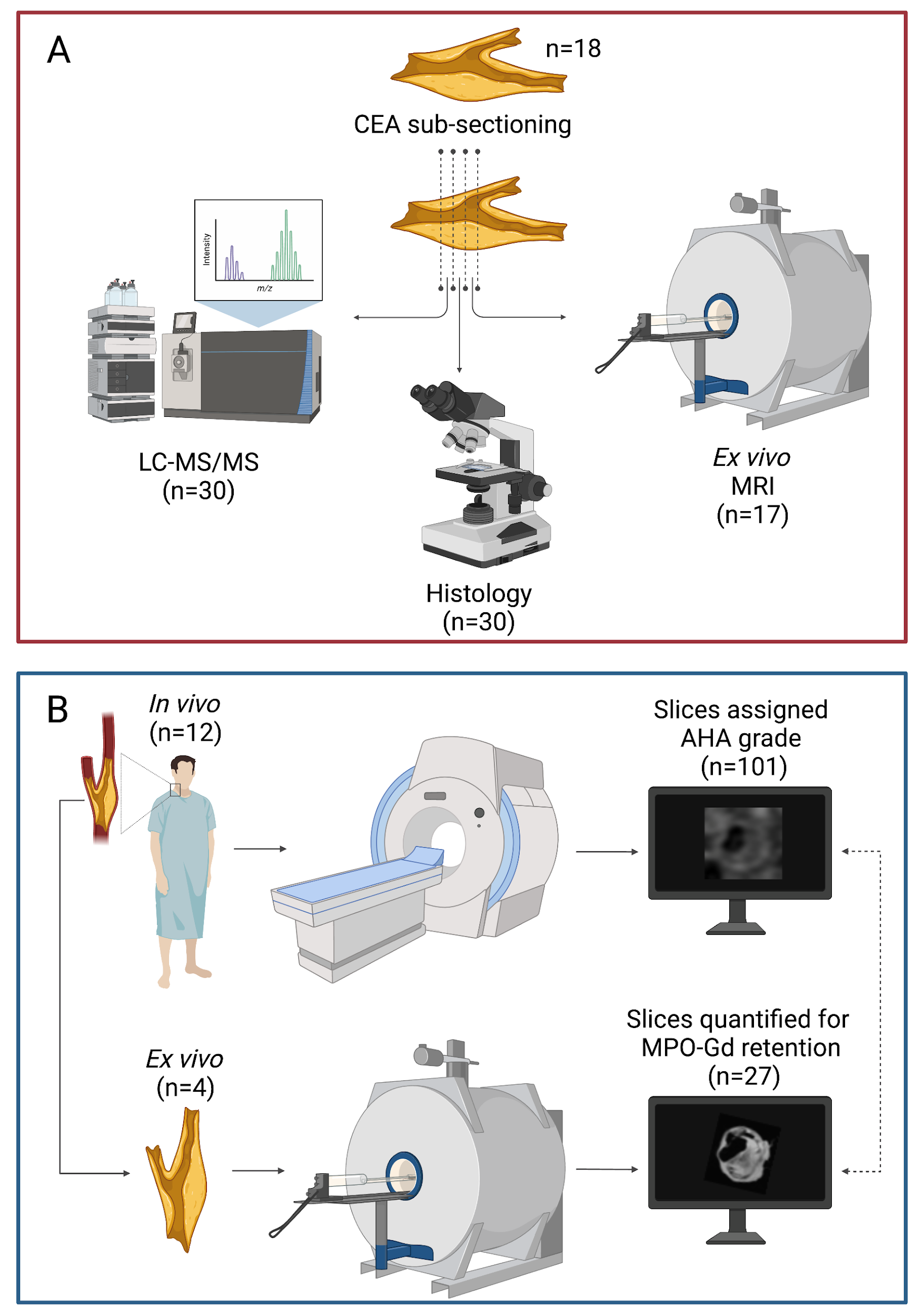
**

**Figure S5**. **Carotid endarterectomy specimen workflow**. Carotid endarterectomy (CEA) specimens were collected from a total of n=30 individual patients. (**A)** CEA specimens from 18 patients were excised and divided into 3-5 mm thick sections. Sub-sections underwent histological validation with adjacent tissues assessed for MPO activity using LC-MS/MS and *ex vivo* molecular MRI using MPO-Gd. From the 18 CEA divided in this manner, histology and LC-MS/MS was performed on 30 sub-sections. *Ex vivo* MRI was performed on 9 CEA sub-sections from 9 separate patients with 2 slices analysed per replicate, except for one case where 1 slice was analysed (n=17). (**B)** From the 30 individual patients, 12 underwent *in vivo* carotid MRI for atherosclerotic plaque characterization and AHA grading within 7 days prior to scheduled surgery. 101 MRI slices were assessed and assigned an AHA grade. Once excised, 4 whole plaques were imaged at baseline and following MPO-Gd activation. *In vivo* and *ex vivo* MRI images were co-registered and MRI-based AHA grading from *in vivo* studies compared with MPO-Gd retention on a slice-by-slice basis (n=27).


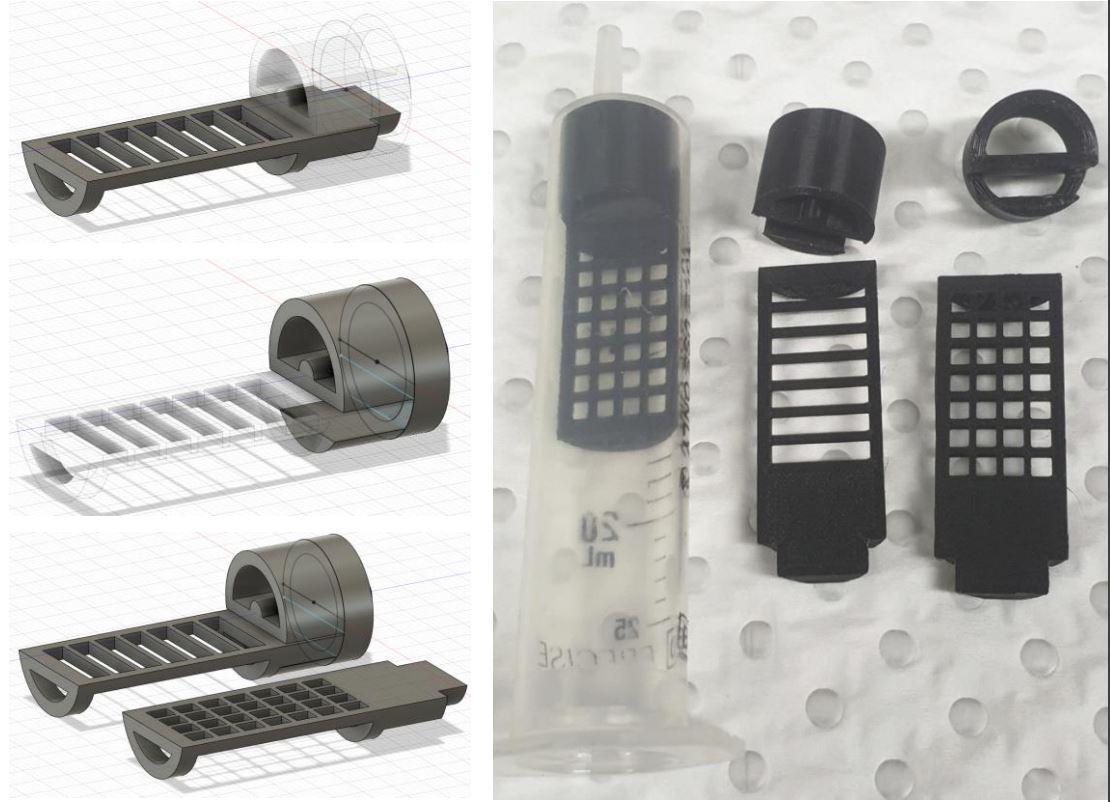


**Figure S6**. **CEA sample holder used for *ex vivo* MRI.** A disposable 3D printed sample holder made up of a grooved spacer and grid was designed to fit a 20 mL syringe. Whole CEA or tissue sub-sections were fixed to the grid with a small amount of glue. The spacer was glued into the base of the syringe and secured to the spacer by the groove. Tissue then underwent soaking, washing, and imaging with respective fluid media, which was kept in place with the syringe’s plunger and cap. This environment resulted in minimal change to the tissue’s position, and the grid provided an external fiducial marker for the co-registration of MRI sequences.


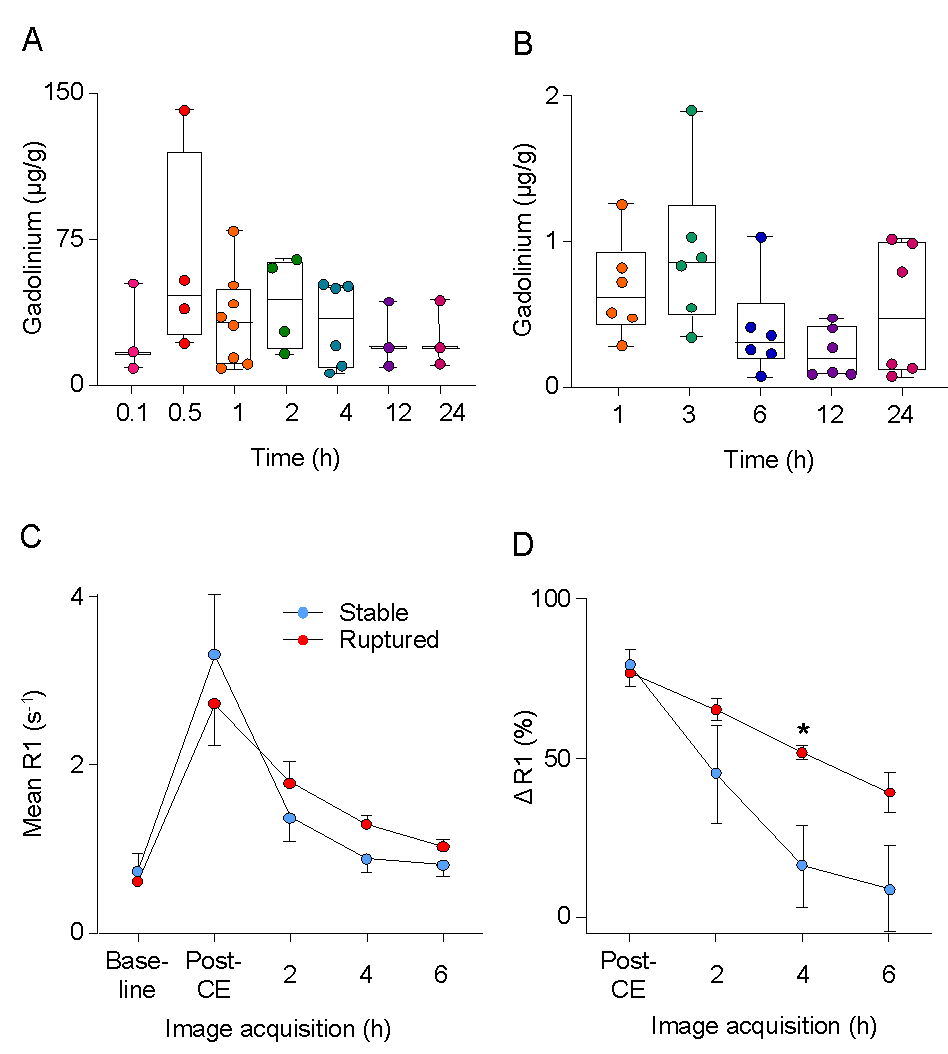


**Figure S7. Optimisation of *ex vivo* MPO imaging for human carotid plaques.** (**A)** Gadolinium-DTPA (DTPA-Gd) soaking experiments. Tissue sections were soaked *ex vivo* in DTPA-Gd and gadolinium quantified by inductively coupled plasma mass spectrometry (ICP-MS). (**B**) DTPA-Gd washing experiment. Tissue sections were soaked in DTPA-Gd for 1 h prior to being washed and gadolinium quantified by ICP-MS. (**C, D)** Tissue-specific DTPA-Gd retention. Histologically confirmed stable and ruptured plaques were soaked with MPO-Gd for 1 h and then washed for the time indicated. Mean R1 and percentage ΔR1 from baseline were then quantified. The 4 h timepoint was selected for the differentiation of ruptured (n=6) from stable (n=8) plaques: relaxation rate R1 for stable (0.9 ± 0.2 s^-1^) and ruptured plaques (1.3 ± 0.1 s^-1^) (*P*=0.0002); R1 percentage change from baseline for stable (16 ± 13%) and ruptured plaques (52 ± 3%) at 4 h (**P*<0.0001). CE; contrast enhancement.
